# *Plasmodium* kinesin-8X associates with mitotic spindles and is essential for oocyst development during parasite proliferation and transmission

**DOI:** 10.1371/journal.ppat.1008048

**Published:** 2019-10-10

**Authors:** Mohammad Zeeshan, Fiona Shilliday, Tianyang Liu, Steven Abel, Tobias Mourier, David J. P. Ferguson, Edward Rea, Rebecca R. Stanway, Magali Roques, Desiree Williams, Emilie Daniel, Declan Brady, Anthony J. Roberts, Anthony A. Holder, Arnab Pain, Karine G. Le Roch, Carolyn A. Moores, Rita Tewari

**Affiliations:** 1 School of Life Sciences, Queens Medical Centre, University of Nottingham, Nottingham, United Kingdom; 2 Institute of Structural and Molecular Biology, Department of Biological Sciences, Birkbeck College, London, United Kingdom; 3 Department of Molecular, Cell and Systems Biology, University of California Riverside, Riverside, California, United States of America; 4 Biological Environmental Sciences and Engineering Division, King Abdullah University of Science and Technology, Thuwal, Jeddah, Kingdom of Saudi Arabia; 5 Nuffield Department of Clinical Laboratory Sciences, University of Oxford, John Radcliffe Hospital, Oxford, United Kingdom; 6 Department of Biological and Medical Sciences, Faculty of Health and Life Science, Oxford Brookes University, Gipsy Lane, Oxford, United Kingdom; 7 Institute of Cell Biology, University of Bern, Bern, Switzerland; 8 Malaria Parasitology Laboratory, The Francis Crick Institute, London, United Kingdom; 9 Research Center for Zoonosis Control, Global Institution for Collaborative Research and Education (GI-CoRE), Hokkaido University, Kita-ku, Sapporo, Japan; Boston College, UNITED STATES

## Abstract

Kinesin-8 proteins are microtubule motors that are often involved in regulation of mitotic spindle length and chromosome alignment. They move towards the plus ends of spindle microtubules and regulate the dynamics of these ends due, at least in some species, to their microtubule depolymerization activity. *Plasmodium spp*. exhibit an atypical endomitotic cell division in which chromosome condensation and spindle dynamics in the different proliferative stages are not well understood. Genome-wide shared orthology analysis of *Plasmodium spp*. revealed the presence of two kinesin-8 motor proteins, kinesin-8X and kinesin-8B. Here we studied the biochemical properties of kinesin-8X and its role in parasite proliferation. *In vitro*, kinesin-8X has motility and depolymerization activities like other kinesin-8 motors. To understand the role of *Plasmodium* kinesin-8X in cell division, we used fluorescence-tagging and live cell imaging to define its location, and gene targeting to analyse its function, during all proliferative stages of the rodent malaria parasite *P*. *berghei* life cycle. The results revealed a spatio-temporal involvement of kinesin-8X in spindle dynamics and an association with both mitotic and meiotic spindles and the putative microtubule organising centre (MTOC). Deletion of the kinesin-8X gene revealed a defect in oocyst development, confirmed by ultrastructural studies, suggesting that this protein is required for oocyst development and sporogony. Transcriptome analysis of *Δkinesin-8X* gametocytes revealed modulated expression of genes involved mainly in microtubule-based processes, chromosome organisation and the regulation of gene expression, supporting a role for kinesin-8X in cell division. Kinesin-8X is thus required for parasite proliferation within the mosquito and for transmission to the vertebrate host.

## Author summary

Kinesins are microtubule-based motors that play key roles in intracellular transport, cell division and motility. Members of the kinesin-8 family contribute to chromosome alignment during cell division in many eukaryotes. However, the roles of kinesins in the atypical cell division of *Plasmodium*, the causative agent of malaria, is not known. In contrast to many other eukaryotes, *Plasmodium* proliferates by endomitosis, in which genome replication and division occur within a nucleus bounded by a persistent nuclear envelope. We show that the *Plasmodium* genome encodes up to nine kinesins and we further investigate the role of kinesin-8X throughout the *Plasmodium berghei* life cycle using biochemical, cellular and gene targeting approaches. We show that *Plasmodium* kinesin-8X has microtubule-based motility and depolymerization activity. We also show that kinesin-8X is probably located on putative microtubule organizing centres (MTOCs) and spindles during cell division in most stages of the life cycle. By gene deletion we demonstrate that kinesin-8X is essential for normal oocyst development and sporozoite formation. Genome-wide RNA analysis of *Δkinesin-8X* gametocytes revealed modulated expression of genes involved in microtubule-based processes. Overall, the data suggest that kinesin-8X is a molecular motor playing an essential role during endomitosis in oocyst development in the mosquito and parasite transmission.

## Introduction

Kinesins are molecular motors that use ATP to translocate along microtubules (MTs) or control MT-end dynamics. There are 14 to 16 classes of kinesins in eukaryotes [1–3] which are defined by their conserved motor domain. This domain contains both ATP and MT binding sites, is located in different contexts within the protein primary sequence, and is required by these motor proteins to undertake a wide range of cellular functions [4]. Many kinesins, together with dynein, have important roles in mitosis, including spindle pole separation, kinetochore attachment to spindles, chromosome alignment and segregation, and cytokinesis [5, 6]. Some of these kinesins have also been shown to play an essential role in meiosis, mainly during meiosis I, where recombination takes place [7–9].

Kinesin-8s are conserved across eukaryotes [2, 3]. During mitosis, kinesin-8 proteins in many eukaryotes localise to spindles and control spindle length and chromosome positioning at the cell equator [10–14]. In the absence of functional kinesin-8, mitotic spindle length increases and chromosome alignment at the metaphase plate is perturbed [10, 15, 16]. In addition, kinesin-8 proteins may have a role in maintenance of cell polarity and nuclear positioning in the centre of a fission yeast [17–19]. At the molecular level, kinesin-8s are plus end directed MT motors that play a key role in controlling MT length, with some exhibiting MT depolymerisation activity [20–22].

Malaria is the most deadly parasitic disease and is caused by the unicellular protozoan *Plasmodium* spp., a genus of Apicomplexa that infects many vertebrates and is transmitted by female *Anopheles* mosquitoes [23]. The parasite has a complex life cycle, alternating between its two hosts. Proliferation occurs by closed endomitotic division, in which genome replication and division occur within a nucleus bounded by a persistent nuclear envelope [24, 25]. Both the replication rate and the number of rounds of division vary between different *Plasmodium* species, at different stages of the life cycle, and within different hosts and host cell types [24, 25]. During asexual stages, nuclear division is asynchronous and is followed by synchronous cytokinesis to produce multiple haploid progeny; this process is called schizogony within vertebrate hepatocytes and erythrocytes, and sporogony within oocysts attached to the mosquito gut basal lamina [25, 26]. Haploid sexual progenitor cells—male and female gametocytes—remain arrested early in the cell cycle within red blood cells. They only produce gametes following ingestion in a blood meal within a mosquito gut, where environmental conditions are optimal for gametocyte activation [27, 28]. Male gametocytes undergo three successive rounds of rapid DNA replication producing an 8N nucleus within 15 minutes of activation, followed by exflagellation to release eight flagellated male gametes [29, 30]. *Plasmodium* lacks a classical centriole to nucleate spindle MTs and drive spindle formation; however, there is evidence of centriolar plaques embedded in the nuclear membrane, which initiates the polymerization of spindle microtubules [31, 32]. This finding has been corroborated recently using live cell imaging with a tagged centrin, *Pb*CEN-4-GFP [33]. As in other eukaryotes, spindle MTs are attached to the sister chromatids and separate them, moving them towards the spindle poles at the end of mitosis [31, 34]. However, the role of kinesins in spindle dynamics during endomitosis in *Plasmodium* has not been studied.

Phylogenetic analyses have identified 9 kinesin genes in the *Plasmodium* genome [2, 3, 35] though not much is known overall about the roles of these motors in Apicomplexa. Here we have analysed a high-resolution representation of the phylogenetic distribution of kinesins across a range of Apicomplexa model organisms including *Plasmodium*. Our findings support earlier studies in identifying two kinesins classified as kinesin-8s which, according to the classification scheme of Wickstead and colleagues [3], are members of distinct kinesin-8 subgroups, kinesin-8B and kinesin-8X. To understand the role of *Plasmodium* kinesin-8X in spindle formation during chromosome separation and the atypical nuclear division, we first analysed the biochemical properties of both *P*. *falciparum* and *P*. *berghei* proteins. We demonstrate that the motor domain is an MT-stimulated ATPase that drives MT gliding and has MT depolymerization activity. Both *P*. *berghei* and *P*. *falciparum* kinesin-8X have these activities. We then analysed kinesin-8X location and function throughout the entire parasite life cycle using the rodent malaria model, *P*. *berghei*. Live cell imaging of *P*. *berghei* showed that kinesin-8X is located on the spindle during mitotic and meiotic division at various stages of the parasite life cycle. Deletion of the gene results in impaired endomitotic process and sporozoite development in the oocyst in mosquitoes, thereby blocking transmission of the parasite to its vertebrate host.

## Results

### Phylogenetic analysis of kinesins in Apicomplexa identifies 15 families, of which nine are present in *Plasmodium*

**Fig 1:**
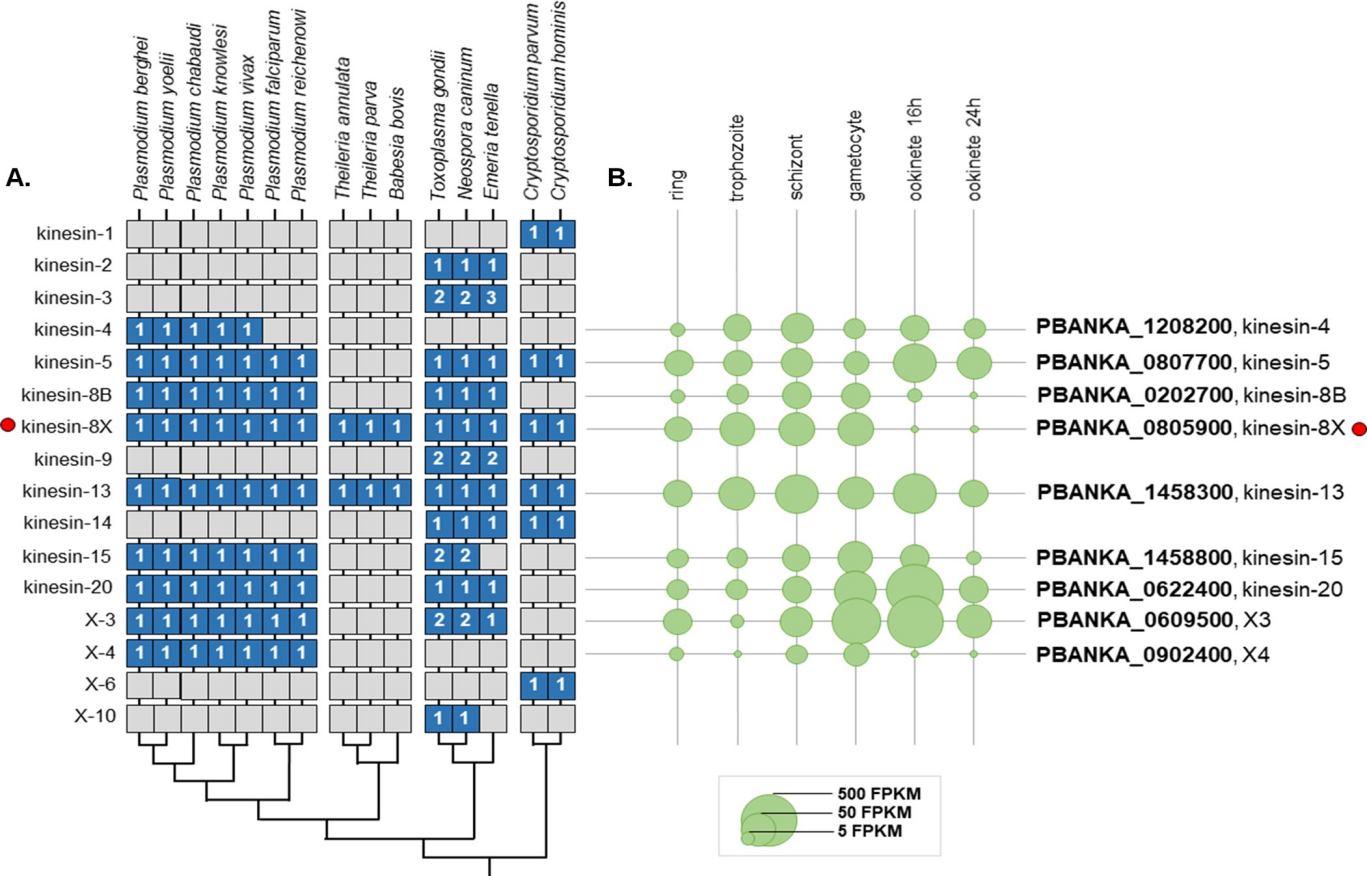
Phylogenetic analysis of apicomplexan kinesins. **(A)** Phylogenetic distribution of detected kinesin genes in alveolate genomes. Blue boxes denote the presence of genes, with the number of detected genes shown. **(B)** The expression levels of *P*. *berghei* genes in different developmental stages [36] are shown as circles. Note that *Plasmodium* spp. contain two kinesin-8 genes.

Using a previously published dataset of kinesin protein sequences as a starting point [3], we conducted a bioinformatic analysis to produce a high-resolution phylogenetic distribution of kinesins across a range of Apicomplexa, focusing on *Plasmodium*. We identified 15 kinesin families in Apicomplexa, nine kinesin genes in *P*. *berghei* and eight in *P*. *falciparum*, including the two kinesin-8 genes in both species (Fig 1, S1 Fig and S1 Table). With the exception of coccidian kinesin-9, kinesin-15, and kinesin-X3, multiple kinesins detected within a single genome all belong to the same orthogroup (http://orthomcl.org/orthomcl/), suggesting that they arose through gene duplication. Kinesin-13s are conserved in evolution across all Apicomplexa clades, suggesting essential roles for these motors, but remarkably, we did not detect kinesin-4 in the Laverania species, *P*. *falciparum* and *P*. *reichenowi*. While kinesin-8B is restricted to Haemosporidia and Cocccidia, kinesin-8X is evolutionary conserved across all Apicomplexa.

### Kinesin-8X motor domain has MT-based motility and depolymerization activities

**Fig 2:**
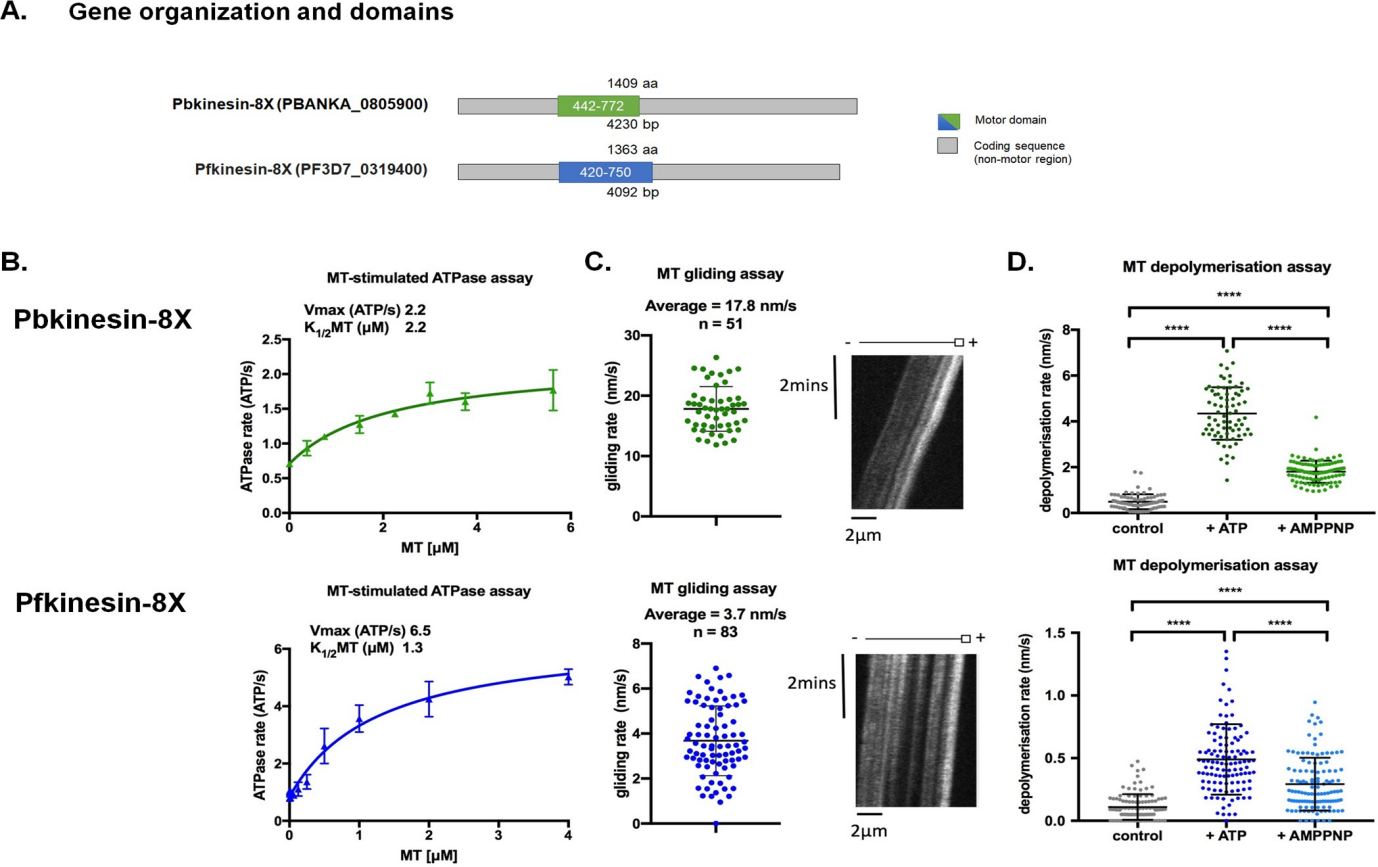
Kinesin-8X shows ATPase, gliding motor and depolymerization activities. **(A)** Schematic protein organisation of PBANKA_0805900 (Pbkinesin-8X) and PF3D7_0319400 (Pfkinesin-8X) showing their full-length sequence and central location of the motor domain, Pbkinesin-8X (green) and Pfkinesin-8X (blue). **(B-D)** Activities of Pb (top—green) and Pf (bottom–blue) kinesin-8X motor domains in three kinesin assays. **(B)** MT stimulated ATPase activity; data fitted to an adapted Michaelis-Menten equation with calculated V_max_, K_m_ and V_0_ parameters. V_0_ was included as a term to aid the curve fitting and account for the non-zero basal ATPase activity of the kinesins in absence of MTs. Error bars represent the mean +/- SD for each MT concentration, n = 3. **(C)** MT gliding activity measured by TIRF microscopy; left, the average motility (nm/s) and individual data points are plotted. The difference between *Pb*kinesin-8X and *Pf*kinesin-8X velocity is statistically significant (t-test P <0.0001). Error bars represent the mean +/- SD; right, an exemplar kymograph demonstrates plus-end directed MT gliding using polarity-marked MTs (schematic above). **(D)** MT depolymerization measured using TIRF microscopy; depolymerization rate (nm/s) in the presence of ATP and AMPPNP is compared to a control in the absence of nucleotide. Error bars present the mean +/- SD, for *Pb*kinesin-8X control n = 77, AMPPNP n = 74, ATP n = 93, and for *Pf*kinesin-8X control n = 112, AMPPNP n = 116, ATP n = 117. An ordinary one-way ANOVA was performed on the depolymerisation data for *Pb*kinesin-8X and *Pf*kinesin-8X separately in Prism to establish the significance of the nucleotide-dependent differences. Significance values are displayed as asterisks, all p-values were <0.0001 (****) comparing control with the presence of AMPPNP or ATP and comparing activity in the presence of AMPPNP or ATP.

We wanted to investigate the roles of kinesin-8Xs in *Plasmodium* with respect to regulation of MT dynamics during cell division. To understand the molecular properties of the proteins encoded by *P*. *berghei* (*Pb)* kinesin-8X (PBANKA_0805900) and its orthologue in *P*. *falciparum* (*Pf*, PF3D7_0319400), we studied the biochemistry of their conserved motor domains. Both motor domains are located in the middle of the protein sequence (Fig 2A) and show 91% sequence identity. We expressed these motor domains as recombinant proteins—referred to below as *Pb*kinesin-8X-MD and *Pf*kinesin-8X-MD, respectively—and characterised their activities.

*Pb*kinesin-8X-MD and *Pf*kinesin-8X-MD exhibit MT-stimulated ATPase activity (Fig 2B), with V_max_ values of 2.2 and 6.5 ATP/s for *Pb*kinesin-8X-MD and *Pf*kinesin-8X-MD, respectively, and with K_1/2(MT)_ values of 2.2 and 1.3 μM, respectively. Both kinesin-8X motor domains drive plus-end directed MT gliding (Fig 2C), with an average velocity of 17.8 ± 3.7 nm/s (n = 51) and 3.7 ± 1.7 nm/s, (n = 83) for *Pb*kinesin-8X-MD and *Pf*kinesin-8X-MD, respectively. Both kinesin-8X motor domains also depolymerize paclitaxel-stabilised MTs (Fig 2D), with MT depolymerization observed in the presence of AMPPNP and ATP, compared to the no nucleotide control (significance confirmed by a one-way ANOVA test). *Pb*kinesin-8X-MD (4.3 ± 1.1 nm/s +ATP, n = 74; 1.8 ± 0.5 nm/s +AMPPNP, n = 93) depolymerised MTs faster than *Pf*kinesin-8X-MD (0.5 ± 0.3 nm/s +ATP, n = 116; 0.3 ± 0.2 nm/s +AMPPNP, n = 117). Depolymerization was faster in the presence of ATP than AMPPNP for both proteins consistent with the requirement for continuous ATPase activity to drive MT depolymerisation.

In summary, we conclude that, like a number of other kinesin-8s [20, 21], *P*. *berghei* and *P*. *falciparum* kinesin-8X motor domains are capable of MT translocation and MT depolymerization *in vitro*, and hence have the potential to regulate MT dynamics *in vivo*.

### Pbkinesin-8X is transcribed at most *P*. *berghei* developmental stages

To understand the context in which these activities could operate, we investigated expression and localisation of kinesin-8X specifically in *P*. *berghei*, by first examining its transcript level using quantitative real time PCR (qRT-PCR) at different developmental stages. The transcription profile showed expression of *Pbk*inesin-8X at most stages of parasite development with the highest RNA level in gametocytes, followed by blood stage schizonts and ookinetes (S2A Fig). These results are comparable to those obtained in previous global RNAseq analyses of *P*. *berghei* [36, 37].

### Spatio-temporal profile of Pbkinesin-8X revealed by live cell imaging of mitotic and meiotic stages in parasite development

To investigate the subcellular location of kinesin-8X in *P*. *berghei*, we generated a transgenic parasite line by single crossover recombination at the 3’ end of the endogenous *kinesin-8X* locus, to express a C-terminal GFP-tagged fusion protein (S2B Fig). PCR analysis of genomic DNA using locus-specific diagnostic primers indicated correct integration of the GFP tagging construct (S2C Fig). The presence of protein of the expected size (~188 kDa) in a gametocyte lysate was confirmed by western blot analysis using GFP-specific antibody (S2D Fig). The kinesin-8X-GFP parasites completed the full life cycle with no detectable phenotype resulting from the GFP tagging (S2A Table).

**Fig 3:**
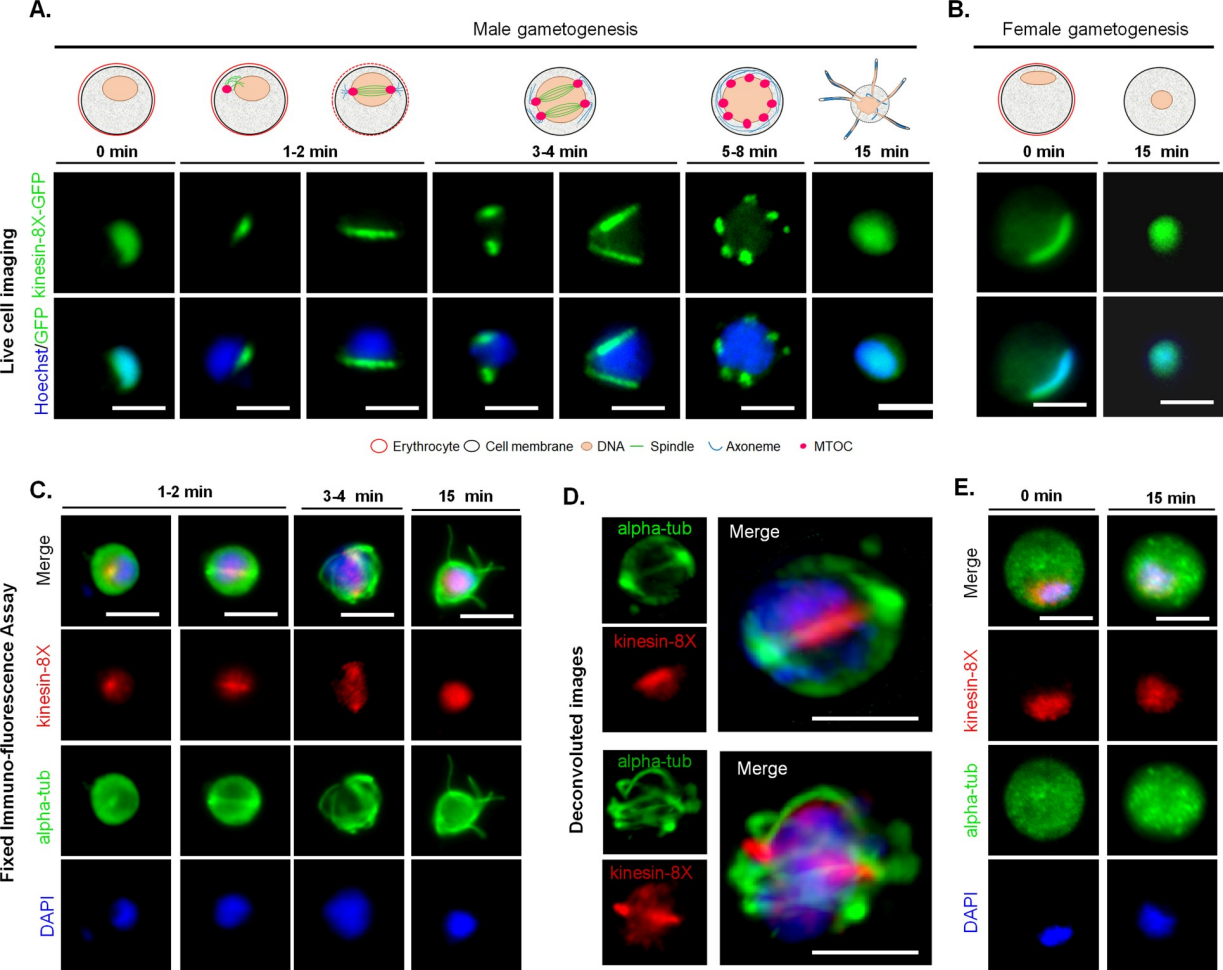
Dynamics of *Pb*kinesin-8X show localization on spindle fibres during male gametogenesis. **(A)** Live imaging of *Pb*kinesin-8X-GFP (green) during male gametogenesis showing an initial location at the putative microtubule organizing centre (MTOC) just after activation, and then on spindles and spindle poles in later stages. **(B)** Localization of *Pb*kinesin-8X (red) during female gametogenesis before (0 min) and after activation (15 min). **(C)** Indirect immunofluorescence assays showing co-localization of *Pb*kinesin-8X (red) and α-tubulin (green) during male gametogenesis. **(D)** Deconvoluted images of male gametocytes showing *Pb*kinesin-8X (red) with α-tubulin (green). **(E)** Indirect immunofluorescence assays showing location of *Pb*kinesin-8X (red) and α-tubulin (green) during female gametogenesis. Scale bar = 5 μm.

The expression and localization of kinesin-8X was assessed by live cell imaging throughout the parasite life cycle. Kinesin-8X was not detectable by microscopy in asexual blood stages (S2E Fig) but exhibited a diffuse nuclear localization in both male and female gametocytes. Following activation of gametogenesis with xanthurenic acid and decreased temperature *in vitro* [28, 38], kinesin-8X began to accumulate in male gametocytes at one end of the nucleus, presumably at the putative MTOC (Fig 3A). Within one-minute of activation we observed an arc-like distribution of kinesin-8X across the nucleus, later forming two distinct foci that is consistent with the formation of two MTOCs (Fig 3A). As DNA replication and endomitosis continued, six to eight distinct foci were seen to form 8 to 10 min after activation (Fig 3A). These kinesin-8X foci may be associated with the MTOCs of the 8N nucleus that precedes exflagellation to produce eight male gametes (Fig 3A). However, there was no detectable expression of Pbkinesin-8X in these male gametes (Fig 3A). To examine further the location of kinesin-8X, we investigated its co-localization with MTs (using α-tubulin as a marker) by indirect immunofluorescence assay (IFA) in fixed cells. Kinesin-8X was localized on MTs during the early stages of male gametogenesis but in later stages it was distributed diffusely within the nucleus (Fig 3C). To improve visualisation, we used deconvolution microscopy and confirmed that kinesin-8X is localized on mitotic spindles in early stages of male gametogenesis (Fig 3D). In female gametocytes there was no major change in kinesin-8X distribution and it remained nuclear even 15 min post-activation (Fig 3B). During this period the nucleus, together with the appearance of kinesin-8X staining, became more condensed and centrally located within the female gamete (Fig 3B).

**Fig 4:**
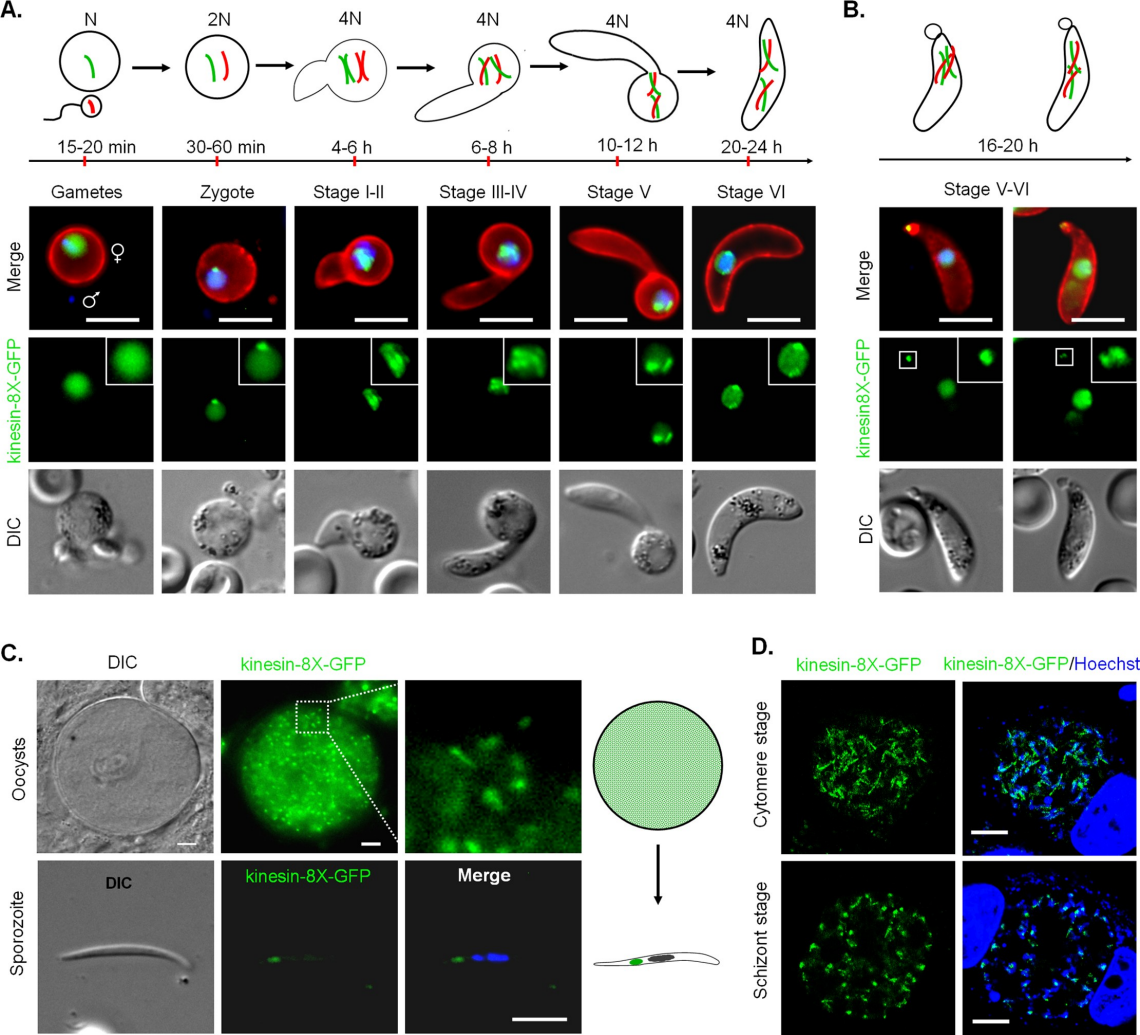
*Pb*kinesin-8X localizes to a putative MTOC and spindle during ookinete development, sporogony and liver stage development. **(A)** Live cell imaging showing *Pb*kinesin-8X-GFP location during ookinete development. A cy3-conjugated antibody, 13.1, which recognises the protein P28 on the surface of activated female gametes, zygotes and ookinetes was used to mark these stages (red). **(B)** Representative images showing *Pb*kinesin-8X, located at basal end of early-mature ookinetes as well as in the nucleus. **(C)** *Pb*kinesin-8X-GFP location in oocyst and sporozoite. **(D)** Location of *Pb*kinesin-8X in liver stages. Scale bar = 5 μm.

Next, we examined the location and dynamics of kinesin-8X during zygote differentiation into the motile ookinete over 24 h [39]. Two hours after fertilisation, kinesin-8X began accumulating at one end of the nucleus, as determined by live cell imaging (Fig 4A). Following the initial protrusion of the apical prominence during stage I and II of ookinete development, kinesin-8X was observed on spindles (Fig 4A). In later stages (stage V), it accumulated at two distinct foci, probably at two spindle poles, and remained there in the mature stages of ookinete development (Fig 4A). Interestingly, in some early mature ookinetes (stage V-VI) some kinesin-8X was located at the basal end of the cell, but it disappeared from this location in fully mature ookinetes (Fig 4B).

Ookinetes traverse the mosquito gut epithelium and develop into oocysts that produce sporozoites. During oocyst development at about 10 to 14-days post-mosquito feeding, kinesin-8X formed punctate dots located near putative MTOCs (Fig 4C). Many arc or bridge-like structures were also observed that may represent the redistribution of kinesin-8X on spindles during this endomitosis in oocysts (Fig 4C). Kinesin-8X was also present in sporozoites, located as a focal point next to the nucleus (Fig 4C). We further analysed kinesin-8X location in oocysts and sporozoites using a recombinant parasite line derived by crossing kinesin-8X-GFP (green) and Ndc80-Cherry (red)-expressing lines (S3 Fig). Kinesin-8X was observed near the nucleus and adjacent to Ndc80, but there was no overlap between the two proteins in either oocyst or sporozoite (S3A and S3B Fig).

To study the expression and location of kinesin-8X during the vertebrate pre-erythrocytic stage, we infected HeLa cells with sporozoites. The pattern of protein location during liver stage development was similar to that in other mitotic stages, with a spindle pattern in cytomere stages and an MTOC-like location in schizont stages (Fig 4D).

### *Pb*kinesin-8X is required for endomitotic division during oocyst development and for parasite transmission

**Fig 5:**
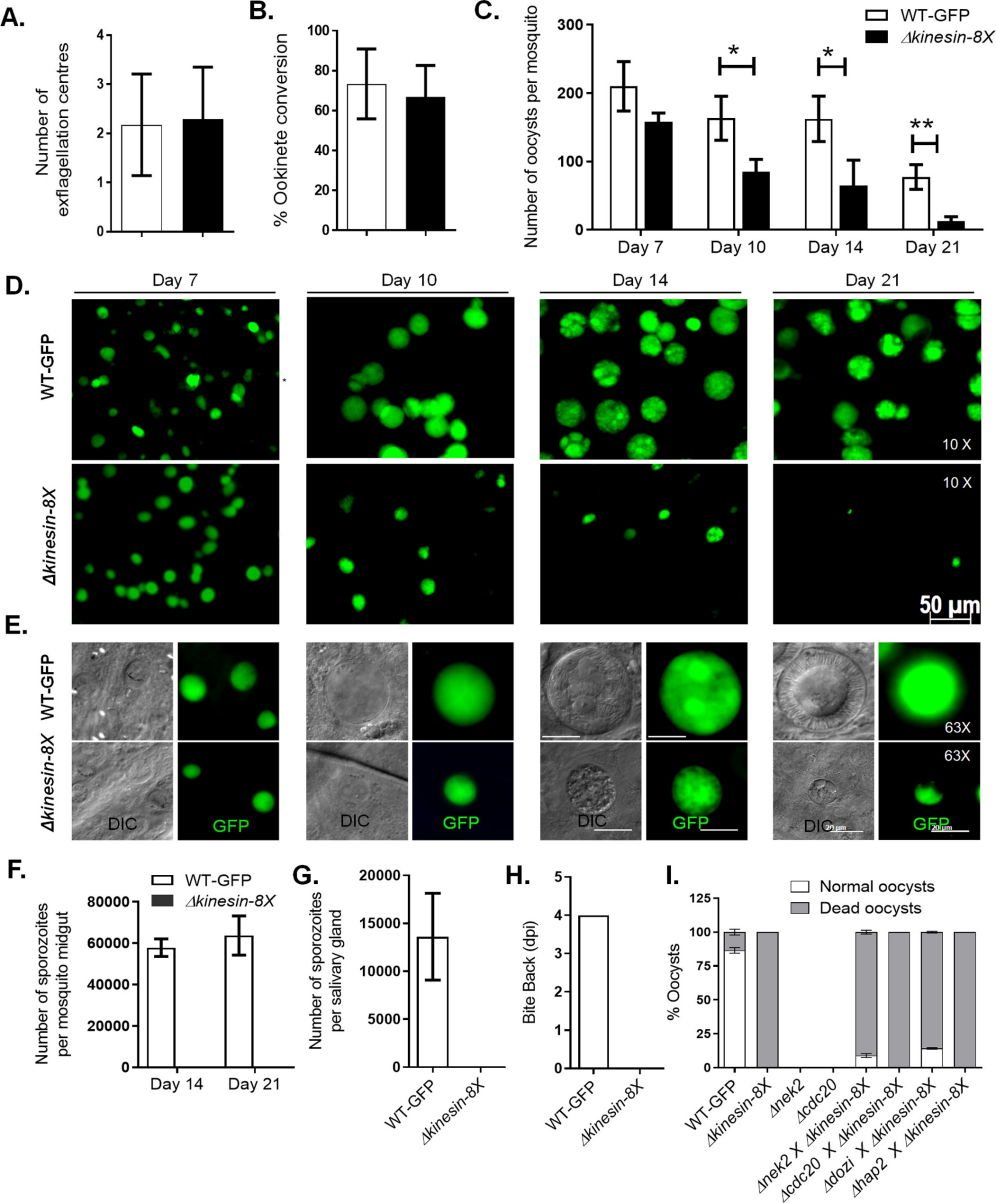
*Pb*kinesin-8X is essential for oocyst development and sporogony. **(A)** Male gametogenesis (exflagellation) of *Δkinesin-8X* line (black bar) compared with WT-GFP line (white bar) measured as the number of exflagellation centres per field. Mean ± SD. n = 5 independent experiments. **(B)** Ookinete conversion as a percentage for *Δkinesin-8X* (black bar) and WT-GFP (white bar) parasites. Ookinetes were identified using 13.1 antibody as a surface marker and defined as those cells that differentiated successfully into elongated ‘banana shaped’ ookinetes. Mean ± SD. n = 5 independent experiments. **(C)** Total number of GFP-positive oocysts per infected mosquito in *Δkinesin-8X* (black bar) compared to WT-GFP (white bar) parasites at 7, 10, 14 and 21-day post-infection (dpi). Mean ± SD. n = 3 independent experiments (>15 mosquitoes for each) *p≤0.05, **p≤0.01. **(D)** Mid guts at 10x magnification showing oocysts of *Δkinesin-8X* and WT-GFP lines at 7, 10, 14 and 21 dpi. Scale bar = 50 μm. * *p* ≤ 0.05 and ** *p* ≤ 0.01 **(E)** Mid guts at 63x magnification showing oocysts of *Δkinesin-8X* and WT-GFP lines at 7, 10, 14 and 21 dpi. Scale bar = 20 μm. **(F)** Total number of sporozoites in oocysts of *Δkinesin-8X* (black bar) and WT-GFP (white bar) parasites at 14 and 21 dpi. Mean ± SD. n = 3 independent experiments. **(G)** Total number of sporozoites in salivary glands of *Δkinesin-8X* (black bar) and WT-GFP (white bar) parasites. Bar diagram shows mean ± SD. n = 3 independent experiments **(H)** Bite back experiments showing no transmission of *Δkinesin-8X* parasites (black bar) where WT-GFP parasites (white bar) show successful transmission from mosquito to mice. Mean ± SD. n = 3 independent experiments **(I)** Rescue experiment showing male allele of *Δkinesin-8X* is affected.

To assess the importance and function of kinesin-8X throughout the *Plasmodium* life cycle, the gene was deleted in *P*. *berghei* using a double crossover homologous recombination strategy (S2F Fig). Diagnostic PCR was performed to confirm successful integration of the targeting construct at the *kinesin-8X* locus (S2G Fig). Analysis of these transgenic parasites by qPCR confirmed complete deletion of the *kinesin-8X* gene (S2H Fig). The successful deletion of the *kinesin-8X* gene indicates that it is not essential during the asexual blood stage, an idea supported by a recent functional profiling of the *Plasmodium* genome at this stage [40]. Phenotypic analysis was then carried out at other developmental stages, comparing two independent *Δkinesin-8X* parasite lines to the wild type (WT-GFP) parasite. Both knockout clones had the same phenotype and data presented here is combined from both clones. We observed no significant difference in exflagellation during male gametogenesis (Fig 5A), and both zygote formation and ookinete development also proceeded successfully (Fig 5B). By light microscopy there were no detectable morphological differences between *Δkinesin-8X* and WT-GFP ookinetes (S4A Fig), and both DNA content (S4B Fig) and gliding motility (S4C Fig, S1V and S2V Videos) of *Δkinesin-8X* and WT-GFP ookinetes were similar suggesting that ookinetes are not affected by the *Δkinesin-8X* deletion.

To assess the effect of the gene deletion on oocyst development, we fed *Anopheles stephensi* mosquitoes on mice infected with *Δkinesin-8X* and wild-type parasites, and the number of GFP-positive oocysts on the mosquito gut wall was counted. There was no significant difference in the number of *Δkinesin-8X* oocysts compared to wild-type controls at 7 dpi (days post-infection), but by 10 dpi we observed a significant reduction in mutant oocysts, which became even more significant at 14 dpi (Fig 5C). By 21 dpi the number of GFP-positive *Δkinesin-8X* oocysts had decreased further to only 8–10% of the WT-GFP number (Fig 5C). We also detected a significant decrease in the size of the *Δkinesin-8X* oocysts at 10, 14 and 21 dpi (Fig 5D). At 21 dpi most of the *Δkinesin-8X* oocysts were dead, with diminished GFP expression and the presence of disintegrated nuclei (Fig 5E). We also observed no viable sporozoites in these oocysts (Fig 5F), and were unable to detect any salivary gland sporozoites (Fig 5G). Whilst mosquitoes were able to transmit the WT-GFP parasite, and blood stage infection was observed in naïve mice 4 days later, mosquitoes infected with *Δkinesin-8X* parasites at the same time failed to transmit this parasite to susceptible mice (Fig 5H).

### Pbkinesin-8X function during sporogony is partially contributed by the male gamete

Since kinesin-8X is expressed in both male and female gametocytes and parasite development is affected after fertilization, we investigated whether the defect is inherited through the male or female gamete. We performed genetic crosses between *Δkinesin-8X* parasites and other *P*. *berghei* mutants deficient in the production of either male *(Δcdc20* and *Δhap2)* or female *(Δnek2* and *Δdozi)* gametocytes. Genetic crosses between *Δkinesin-8X and Δnek2* or *Δdozi* female mutants produced some normal sized oocysts that were able to sporulate, showing a partial rescue of the *Δkinesin-8X* phenotype (Fig 5I). On the other hand, crosses between *Δkinesin-8X and Δcdc20* or *Δhap2* male mutants showed no rescue of the *Δkinesin-8X* phenotype (Fig 5I). These results indicate that a functional kinesin-8X gene copy inherited from the male is an important, but not an absolute, requirement for oocyst development.

### Ultrastructure of Δ*kinesin-8X* oocysts shows defects in growth and sporozoite budding

**Fig 6:**
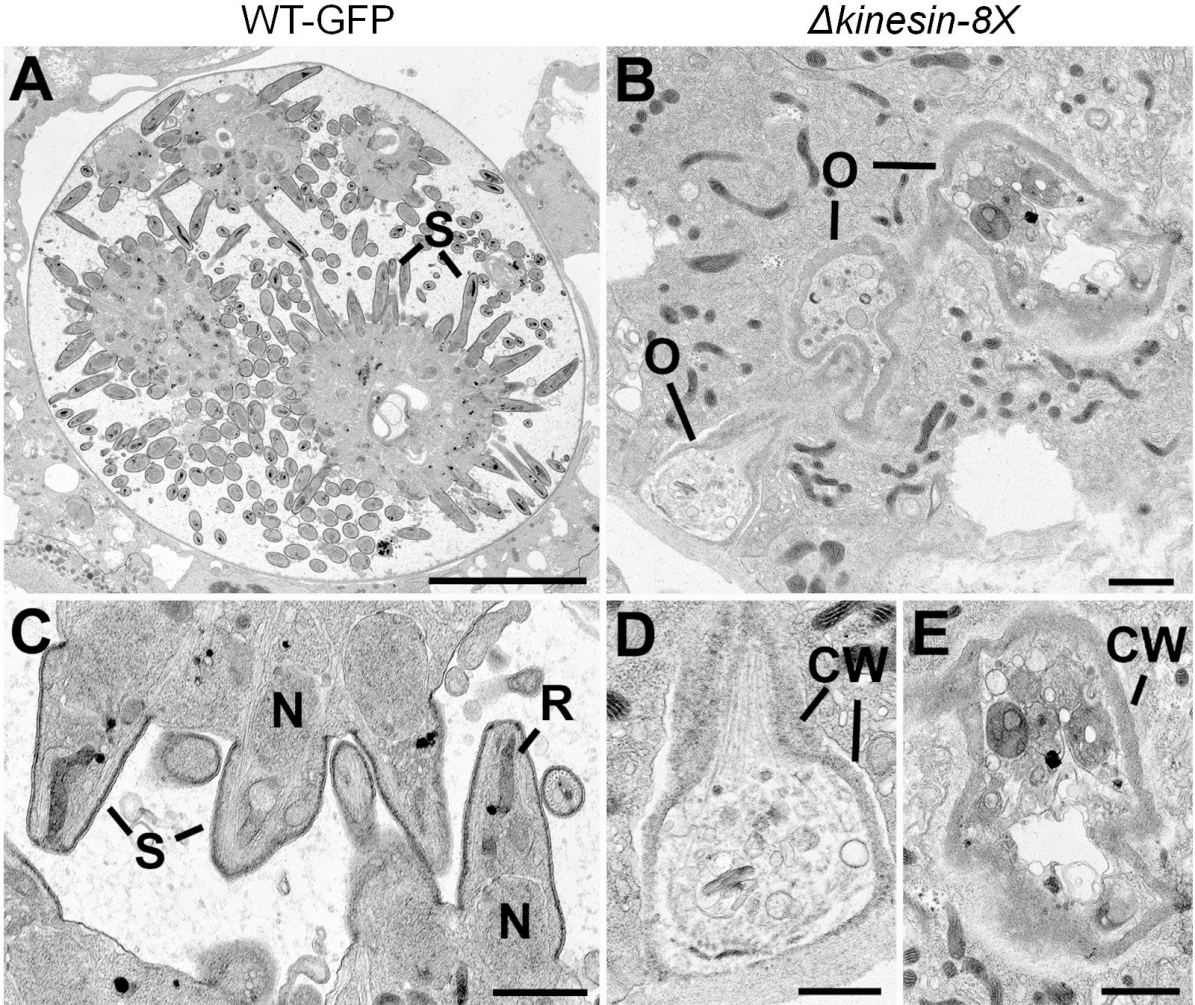
Ultrastructure analysis of oocyst development in Δ*kinesin-8X* parasites. Electron micrographs of WT (A, C) and mutant (B, C, D) oocysts located in the mid gut of the mosquito at 14 days post-infection. Bar is 10 μm in panel A, and 1μm in other micrographs. (A) Low power image through a mid-stage oocyst showing early stages in sporozoite (S) formation. (B) Low power showing the collapsed appearance of three oocysts (O). (C) Detail of an oocysts showing budding sporozoites (S) containing nucleus (N) and developing rhoptry (R). (D, E) Enlargements of oocysts in panel B showing the collapsed oocyst wall (CW) surrounding cytoplasm with degenerate organelles.

To define further the defect in oocyst development at 14 dpi resulting from the absence of kinesin-8X, midguts of both Δ*kinesin-8X* and WT-GFP parasite-infected mosquitoes were examined by transmission electron microscopy. Numerous WT-GFP oocysts were observed at various stages of sporozoite development (Fig 6A), with large numbers of sporozoites budding from the cytoplasmic masses (Fig 6A and 6C). In contrast, it was extremely difficult to identify any apparently healthy Δ*kinesin-8X* oocysts (Fig 6B), and a detailed search identified a few collapsed oocysts with degenerate cytoplasmic organelles (Fig 6B, 6D and 6E).

### Transcriptome analysis of Δ*kinesin-8X* parasites reveals modulated expression of genes involved in motor activity and several other functions

**Fig 7:**
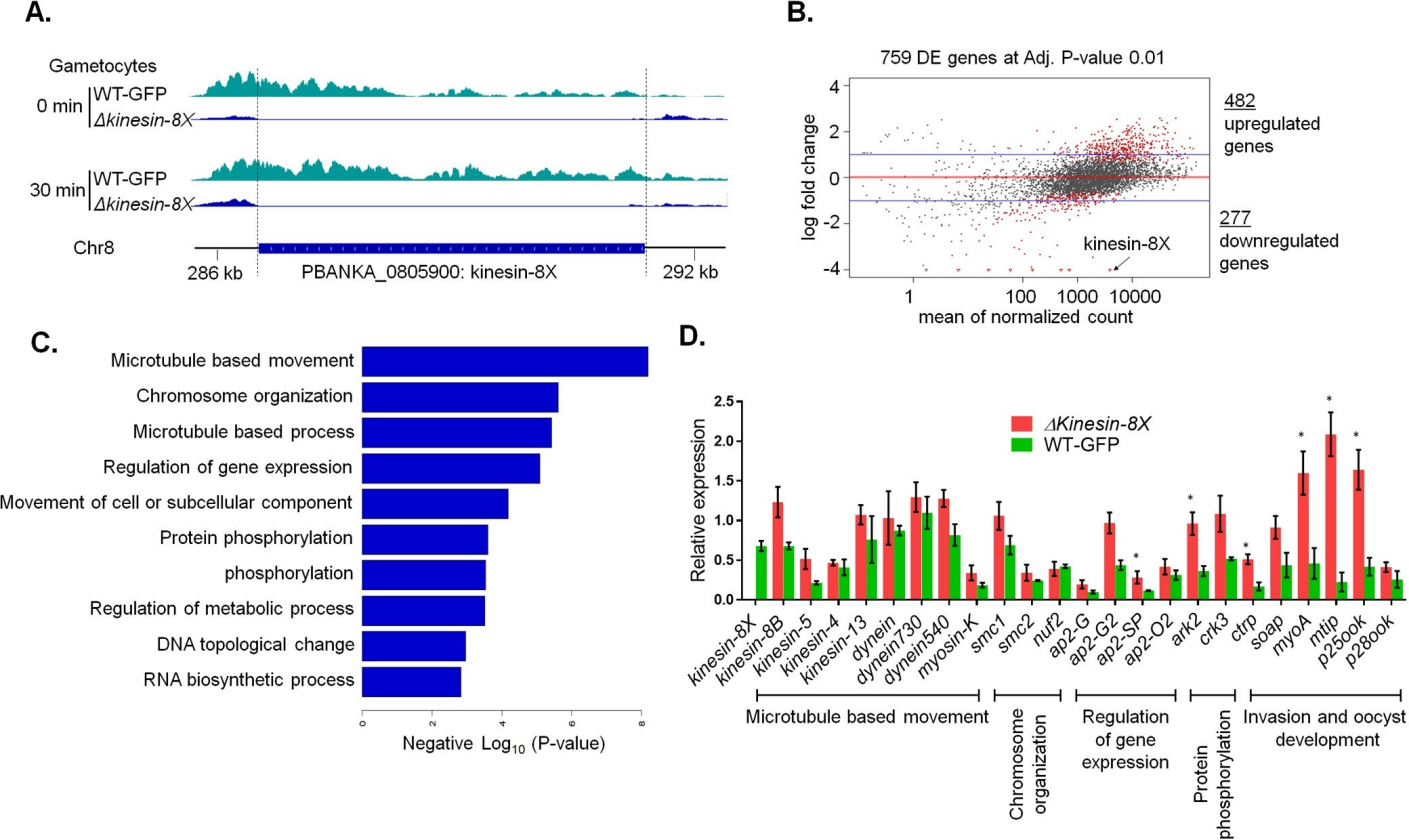
Global transcript analysis of *Δkinesin-8X* parasites by RNAseq. **(A)** RNA sequence analysis showing no transcript in *Δkinesin-8X* parasites. **(B)** Upregulated and downregulated genes in *Δkinesin-8X* parasites compared to WT-GFP parasites. **(C)** Gene ontology enrichment analysis showing most affected genes involved in various biological processes. **(D)** Validation of relevant and selected genes from the RNAseq data by qRT-PCR. Mean ± SD. n = 3 independent experiments. *p ≤ 5.

Although the *Δkinesin-8X* defect was most evident in oocyst development, the fact that it was partially inherited through the male gamete led us to analyse mRNA expression in both *Δkinesin-8X* and WT-GFP gametocytes. Global transcription was investigated by RNAseq analysis immediately before gametocyte activation (0 min) and after exflagellation (30 min after activation). The genome-wide read coverages for the four pairs of biological replicates (WT, 0 min; WT, 30 min; *Δkinesin-8X*, 0 min; and *Δkinesin-8X*, 30 min) exhibited Spearman correlation coefficients of 0.97, 0.98, 0.95 and 0.95; respectively, validating the reproducibility of the experiment. The *kinesin-8X* deletion was confirmed in the RNAseq dataset by the lack of reads for this locus (Fig 7A). Furthermore, in total, 482 genes were upregulated, and 277 genes were downregulated in comparison with the WT-GFP control (Fig 7B, S3 Table).

Gene ontology enrichment analysis of the upregulated genes identified genes involved in MT-based processes—including MT-dependent motors—together with other functions including cell division and chromosome organization, indicating a possible mechanism of compensation during cell division for the loss of *kinesin-8X* (Fig 7C). Six of the top fifty, including three of the top twenty most upregulated genes in the *Δkinesin-8X* parasite encode putative dynein heavy chains, consistent with a strong increase in expression of compensatory MT-related motor proteins and a specific association between dynein- and kinesin-driven processes (S3 Table).

Among the downregulated genes, the PIR family was strongly represented, with 57 genes significantly downregulated (S3 Table). Of these PIR genes, eight were described as male gametocyte specific in recent single cell transcriptomics studies [41]. We also identified 24 sub-telomeric genes belonging to the Fam-A, Fam-B and Fam-C families as significantly downregulated (S3 Table), although, it is currently unclear how either the PIR or Fam family genes relate to kinesin-8X function. PIR (Plasmodium interspersed repeat) and Fam (Fam-A, -B,-C) are multigene families, present within the telomeric and sub-telomeric regions of most chromosomes of, *Plasmodium*. There is little known about the function of the proteins encoded by these genes, but existing experimental evidence suggest that they are exported to the host erythrocyte and have a role in immune evasion via a process of differential gene expression and antigenic variation of the proteins [36, 42, 43]. Pertinent to our work, a recent single cell transcriptomics study [41] showed that some of the PIR genes are specifically expressed in male gametocytes, while Fam genes are specifically expressed in female gametocytes. In our study two Fam genes, specifically expressed in female gametocytes, were downregulated while many PIR genes were significantly downregulated further supporting the idea that kinesin-8X is at least partially linked to male gametocyte function.

Differences in transcript levels revealed by RNAseq analysis were further validated by qRT-PCR, focusing on genes involved in motor activity, ookinete invasion and oocyst development (Fig 7D). These data showed a good correlation with the RNAseq results (Fig 7D) further validating the notion of potential compensatory mechanisms in these particular pathways.

## Discussion

Accurate chromosome segregation is vital for all eukaryotes, and elucidating the mechanistic contributions of spindle regulatory factors sheds light on how this achieved. The importance of kinesin-8 molecular motors in mitosis in many eukaryotes is well-established. These motors move on spindle MTs towards their plus ends and regulate spindle dynamics, contributing to spindle length and positioning, thus facilitating chromosome alignment during metaphase [44–46]. However, the exact molecular bases of these cell division functions remain controversial—kinesin-8s have been suggested to have MT stabilising, destabilising, growth slowing, and/or actively depolymerizing activities in spindle function, perhaps according to their specific cellular environment [21, 22, 47].

Using phylogenetic analysis, we identified 15 kinesin families in Apicomplexa, of which 9 are encoded in most *Plasmodium* genomes. Our current work supports the previous finding that the genome of *Plasmodium* contains two kinesin-8 proteins—kinesin-8B and kinesin-8X [3]. We wanted to understand the properties and function of kinesin-8X, which is found across all the investigated apicomplexan parasite but is more distantly related to kinesin-8s that have been characterised in other eukaryotes. The divergence in kinesin repertoire among eukaryotes that this analysis captures could either reflect sequence diversity among kinesin proteins, or underline the plasticity of kinesin functions during the evolution of apicomplexan genomes.

The conserved kinesin motor domain of Pbkinesin-8X, and its orthologue Pfkinesin-8X is located in the middle of each protein, in contrast to its N-terminal location in kinesin-8s of most other organisms [44]. Although this difference in the context of the motor domain and its sequence divergence could influence its properties, we showed that recombinant motor domains from these proteins have a number of activities in common with, for example, the human kinesin-8A Kif18A. *Pb*kinesin-8X-MD and *Pf*kinesin-8X-MD are MT-stimulated ATPases and can drive plus-end directed MT gliding [48]. Such gliding activity of motors in an N-terminal location usually indicates that a dimeric full-length kinesin can take multiple steps along MTs. Whether or not the central location of the motor domain modifies the behaviour or function of the full-length *Plasmodium* proteins will be the topic of future studies. The *Plasmodium* kinesin-8X motor domains also showed MT depolymerisation activity, a characteristic shared with kinesin-8s from yeast and humans, and which is important for their MT length regulatory activities [10, 20, 21, 48]. While the MT-stimulated ATPase activity of Pbkinesin-8X is lower than that of Pfkinesin-8X, its ATP-dependent gliding activity and ATP-dependent rate of MT depolymerisation are faster. This is intriguing given the motor domain protein sequences of Pbkinesin-8X and Pfkinesin-8X are 91% identical, and suggests that even these small differences may influence these proteins’ stability or activity. Characterising these differences further will also be an important area for investigation in the future. Pbkinesin-8X and Pfkinesin-8X-catalysed MT depolymerisation is faster in the presence of ATP than in the presence of AMPPNP; this differentiates them from human kinesin-8 [48] and could be indicative of differences in depolymerisation mechanism between kinesin-8s in these species. However, the overall biochemical properties of the Plasmodium kinesin-8X motor domains are consistent with the idea that these proteins combine activities of stepping and MT dynamics control, to ensure accurate cell division in these parasites.

In *P*. *berghei* parasites, kinesin-8X is located near the putative MTOC and on the spindle fibres in most of the proliferative stages found within the mosquito vector including male gametogenesis, ookinete differentiation and sporogony. Furthermore, the dynamic localization of kinesin-8X during male gametogenesis mirrors spindle MT dynamics during chromosome movement and segregation in the three rounds of endomitosis. Consistently, while Pbkinesin-8X and α-tubulin showed some co-localisation in the nucleus, Pbkinesin-8X is confined to the nucleus and this suggests kinesin-8X has a spindle-based role during chromosome dynamics and segregation at this life cycle stage. A similar location and role in MT dynamics has been shown in fission yeast [49], budding yeast [50], Drosophila [51], and humans [10] where kinesin-8 proteins are co-localized with MTs during cell division.

The discrete foci of kinesin-8X during zygote development and differentiation suggest it is associated with the putative MTOC at this stage, when the genome is replicated from 2N to 4N. This is the stage of parasite development when meiosis and genetic recombination occur [52], and after the succeeding oocyst formation, finally giving rise to haploid sporozoites. The spatio-temporal profile of kinesin-8X during the zygote to ookinete transition (stage I-VI) suggests a role in chromosome dynamics and segregation during meiosis. The additional location of kinesin-8X at the basal end of early mature ookinetes (stage V-VI) and its disappearance in fully mature ookinetes is also consistent with a role in movement of the nucleus from the cell body to the ookinete. Subpellicular MTs help in positioning the nucleus within the ookinete in *Plasmodium* but the mechanism by which the nucleus is moved from cell body to ookinete is unknown [53]. The mature ookinete crosses the mosquito gut epithelium and at the basal side of the gut wall it rounds up and develops into an oocyst. Endomitosis in the oocyst consists of many rounds of nuclear division and results in production of hundreds of sporozoites [31, 54]. The distinct kinesin-8X-GFP foci in early stage oocysts and the localization to the MTs suggest that these foci are associated with MTOCs and the bridges are the spindles. This suggestion is supported by our co-localization study with Ndc80 and earlier EM studies of oocysts, in which hemi-spindles and MTOCs were found in the developing oocyst stage [31, 54, 55].

Expression of kinesin-8X in the pre-erythrocytic liver stages suggests that it is also involved in this endomitotic stage of parasite development, although surprisingly it is apparently not present in blood stage schizogony. This is fascinating because it suggests that *Plasmodium* employs other motors in the mitotic spindle at this stage of the life cycle. However, all together our localization data suggest an important role for kinesin-8X in the regulation of MT dynamics during spindle formation and chromosome segregation in endomitosis and meiosis during several asexual and sexual stages. It is also likely that other kinesins can compensate for the loss of Pbkinesin-8X in the life cycle stages–erythrocytic phase, male gametogenesis, ookinete development—in which it is not essential. Our demonstration that upregulation of kinesin-8B and kinesin-13 in the Δ*kinesin-8X* parasite might suggest that one or the other or both of these molecular motors can compensate for the loss of its activity at most stages of the life cycle [3]. Kinesin-13s in particular are another family of well-characterised regulators of microtubule dynamics [56, 57], and the *Plasmodium* kinesin-13 motor domain has previously been shown to have MT depolymerization activity in vitro [58].

Despite a lack of a distinct phenotype at other stages, oocyst formation and maturation were impaired in *Δkinesin-8X* parasites, producing fewer oocysts of smaller size when compared to WT-GFP parasites, in the mosquito gut. Examination of *Δkinesin-8X* oocysts in the midgut by electron microscopy at 14 days revealed non-viable parasites with no evidence of nuclear division or the initiation of sporozoite formation. This is in contrast WT-GFP parasites that exhibited oocysts with a highly lobed syncytial nucleus, associated with formation of large numbers of sporozoites. This suggests that kinesin-8X is involved in the differentiation of invasive ookinetes to oocysts and the endomitotic process during sporogony. A similar phenotype was observed in our recent study on *Plasmodium* specific P-type cyclin PbCYC3 during sporogony [59], in which oocyst size and sporozoite formation are affected. Similar results were observed with other gene deletion mutants including MISFIT [60], PK7 [61], DMC1 [62] and PPM5 [63]. The partial compensation of the Δ*kinesin-8X* phenotype with a female line mutant (*Δnek2 and Δdozi)* indicates that the male lineage may be partially affected in the parasite. This closely resembles what was observed for *Δmisfit* and *Δppm5*, both of which have an absolute requirement for a functional gene from the male line [60, 63]. However, in our *Δkinesin-8X* line, the male contribution seems to be partial. In a genome-wide transcript analysis of the *Δppm5* phosphatase-deficient line, kinesin-8X was upregulated in activated gametocytes [63], suggesting that PPM5 may regulate the activity of kinesin-8X by dephosphorylation. The morphology and DNA content of Δ*kinesin-8X* ookinetes is not different from that of WT-GFP parasites, as was also observed for *Δppm5* parasites, and in contrast to *Δmisfit* parasites in which the ookinete DNA content was less than in WT-GFP [60, 63].

One reason for fewer Δ*kinesin-8X* oocysts may be a defect in ookinete invasion of the mosquito gut wall, as shown recently for *Δppl4*, a deletion of the *Plasmodium* perforin-like protein-4 gene [64]. Interestingly, the expression of PPL4 was downregulated up to 50% in Δ*kinesin-8X* parasites. However, this explanation was ruled out by the ookinete motility and oocyst numbers in mosquitoes at an early stage (day 7) infected with *Δkinesin-8X* parasites, which showed no significant difference from WT-GFP parasites. These findings and the ultrastructural analysis of oocysts, in conjunction with the data on the expression and localization of kinesin-8X, suggest that this kinesin has crucial roles during endomitosis in oocyst development. Global transcript and qPCR analyses of the *Δkinesin-8X* parasites showed significant differential gene expression between the knockout and WT lines. The genes for which the expression was most affected in Δ*kinesin-8X* parasites are involved in MT-based movement. An in-depth analysis of these genes showed upregulation of several kinesins, including *kinesin-8B*, *kinesin-13* and *kinesin-5*, suggesting that their activity might compensate for the loss of *kinesin-8X*. Our recent study showed that the deletion of kinesin-8B affects only axoneme assembly during male gamete (flagellum) formation but the current data indicates that its activity may be repurposed [65]. Several other genes involved in transcriptional regulation such as AP2 transcription factors (e.g. AP2-O2, AP2G2, AP2SP) or genes involved in invasion or oocyst development were significantly upregulated, however it is important to highlight that modulation of the expression of these genes was not able to recover the critical role of *kinesin-8X* during ookinete and oocyst development.

In conclusion, our work reveals that kinesin-8X is associated with both mitotic and meiotic spindles during atypical cell division in most proliferative stages of the life cycle with the notable exception of the asexual blood stages. Most importantly, we validated the essential role of kinesin-8X during endomitosis in oocyst development, indicating that specific inhibition of kinesin-8X could be developed in novel transmission blocking strategies against these devastating parasites.

## Materials and methods

### Ethics statement

The animal work performed in the UK passed an ethical review process and was approved by the United Kingdom Home Office. Work was carried out under UK Home Office Project Licenses (40/3344 and 30/3248) in accordance with the United Kingdom ‘Animals (Scientific Procedures) Act 1986’ and in compliance with ‘European Directive 86/609/EEC’ for the protection of animals used for experimental purposes. Experiments performed in Switzerland were conducted in strict accordance with the guidelines of the Swiss Tierschutzgesetz (TSchG; Animal Rights Laws) and approved by the ethical committee of the University of Bern (Permit Number: BE109/13). Six-to-eight week old female Tuck-Ordinary (TO) (Harlan) outbred mice were used for all experiments in the UK. Balb/c female mice between six and ten weeks of age were used in experiments in Switzerland. Mice were either bred in the central animal facility of the University of Bern, or were supplied by Harlan Laboratories or Charles River Laboratories.

### Phylogenetic analysis of apicomplexan kinesins

The Hidden Markov Model (HMM) model for the kinesin motor domain (PF00225) was downloaded from Pfam (http://pfam.xfam.org/) [66] and searched against a range of apicomplexan genome-encoded protein data sets (S1A Table). As in the previous study [3], a preliminary threshold was set to 1e^-25^. To search for additional apicomplexan kinesins with divergent domains, we employed an additional search strategy using previously published kinesin sequences retrieved from *P*. *falciparum*, *T*. *annulata*, *T*. *gondii*, *C*. *parva*, and *T*. *thermophila* [3]. Reciprocal best BLASTP hits for these kinesins were obtained from an extended set of alveolate protein sets (S1A Table), and for each kinesin the corresponding sequences were collected. Sequences were aligned with mafft [67] and trimmed using trimAl [68]. Lineage-specific HMMs were then built using HMMer (http://hmmer.org/) and used to search the apicomplexan protein sets. To remove false-positives, a simple neighbour-joining tree was constructed (clustalW) from all proteins with detected domains, and all pair-wise genetic distances were calculated. For each protein, the average genetic distance to all other candidates for the same kinesin was compared to the average distance calculated individually for all other types of kinesin proteins. Only if the average distance to the same type of kinesin (for example, the average distance between a kinesin-1 candidate and all other kinesin-1 proteins) was lower than for all other kinesin types, were candidates retained. We refer to these two approaches as 'direct HMM' and 'lineage-specific HMM', respectively.

The direct HMM approach detected a group of *Plasmodium* and coccidian proteins with kinesin motor domains that showed no clear association to previously reported kinesins [3]. These proteins are not included in the presented sets but are listed in S1B Table as 'kinesin-like'. A maximum likelihood tree of the detected kinesin proteins is shown in S1 Fig.

A total of 124 kinesins were detected from the direct HMM approach, and 112 of these (90%) were also identified through the lineage-specific HMM approach. The only additional proteins that were detected by the lineage-specific approach was a group of orthologous zinc-finger proteins (PBANKA_1351200, PY17X_1356300, PCHAS_1355800, PKNH_1263900, PVX_082800, PF3D7_1337400, PRCDC_1336400, TA20465, TP01_0517, BBOV_III002540, TGME49_269940, NCLIV_036650) that showed sequence similarity to X5 kinesins from the Ciliate, *T*. *thermophila*. This group was deemed not to represent *bona fide* kinesins, as none of these proteins contained a kinesin motor domain and sequences were divergent from all other kinesins.

### Recombinant protein expression and purification

#### *P*. *berghei* kinesin-8X recombinant protein expression and purification

DNA sequences to express *P*. *berghei* kinesin-8X motor domain (residues 432–784, termed Pbkinesin-8X-MD) and an equivalent C-terminal SNAPf–tagged construct (Pbkinesin-8X-MD-SNAP) were cloned into a pNIC28-Bsa4 vector (Structural Genomics Consortium) including a TEV-cleavable N-terminal His_6_ tag using LIC cloning. The sequence was verified and the plasmid was transformed into *E*. *coli* BL21*(DE3) for protein expression.

Bacteria were grown at 37°C until an OD_600_ of 0.7 and then switched to 20°C for 30 min before addition of IPTG to 0.5 mM to induce protein expression. After 12 h, cells were harvested and resuspended in lysis buffer (20 mM Tris-HCl pH 7.5, 500 mM NaCl, 5 mM MgCl_2_ and 1 mM ATP with EDTA-free protease inhibitor (Roche)). The cell suspension was sonicated for 45 min and then centrifuged at 48,384 g, 4°C for 1 h. The resulting supernatant was incubated with Ni-NTA agarose resin with mixing at 4°C for 1 h followed by washing with lysis buffer to reduce non-specific binding. The His_6_-Pbkinesin-8X-MD constructs were eluted with lysis buffer containing 100 mM Imidazole and incubated with TEV protease for 12 h to remove the tag. The protein was then exchanged by dialysis to low-salt ion exchange buffer (20 mM Tris-HCl pH 7.5, 100 mM NaCl, 5 mM MgCl_2_) and Ni-NTA resin was used to remove the His_6_-TEV protease from, the flow-through that contained Pbkinesin-8X-MD without the His_6_-tag. Further purification was performed using a 1 ml HiTrap Q HP anion exchange column to remove any residual bacterial protein contaminants from the Pbkinesin-8X-MD that did not bind. The Q column flow-through was concentrated, aliquoted and snap-frozen until further use.

#### *P*. *falciparum* kinesin-8X recombinant protein expression and purification

DNA sequences to express *P*. *falciparum kinesin-8X* motor domain (residues 420–762, termed Pfkinesin-8X-MD) and an equivalent C-terminal SNAPf–tagged construct (Pfkinesin-8X-MD-SNAP) were cloned into a pNIC-CTHF vector (Structural Genomics Consortium) that includes a TEV-cleavable C-terminal His_6_-FLAG tag using Gibson cloning. The DNA sequence was verified and the plasmid transformed into BL21*(DE3) *E*. *coli* cells for protein expression.

Bacteria were grown at 37°C until the OD_600_ was around 0.6 to 0.8, then the culture was cooled to 18°C before addition of IPTG to 0.1 mM to induce protein expression. After 12 h, cells were harvested and resuspended in lysis buffer (50 mM Tris-HCl pH 7.0, 400 mM NaCl, 2 mM MgCl_2_, 1 mM ATP, 2 mM β-mercaptoethanol, 15 μg/ml DNase I (Sigma) with EDTA-free protease inhibitor (Roche). Cells were lysed using an Avesti Emulsiflex C3 high-pressure homogeniser, passaging the lysate three times. The lysate was centrifuged at 48,384 g, 4°C for 1 h and the resulting supernatant was incubated with Ni-NTA agarose resin with mixing at 4°C for 30 min followed by washing with low-imidazole containing buffer (50 mM Tris-HCl pH 7.0, 400 mM NaCl, 2 mM MgCl_2_, 1 mM ATP, 2 mM β-mercaptoethanol, 10 mM imidazole) to reduce non-specific binding. The His_6_-Pfkinesin-8X-MD proteins were eluted with high-imidazole containing buffer (50 mM Tris-HCl pH 7.0, 400 mM NaCl, 2 mM MgCl_2_, 1 mM ATP, 2 mM β-mercaptoethanol, 250 mM Imidazole pH 7.0). Eluted fractions were dialysed for 12 h at 4°C against low-salt buffer (50 mM Tris-HCl pH 7.0, 40 mM NaCl, 2 mM MgCl_2_, 1 mM ATP, 2 mM β-mercaptoethanol) together with TEV protease to remove the C-terminal His_6_-FLAG tag. Dialysed protein was loaded onto a 1 ml HiTrap SP HP cation exchange chromatography column and eluted with gradient to a high-salt buffer (50 mM Tris-HCl pH 7.0, 1 M NaCl, 2 mM MgCl_2_, 1 mM ATP, 2 mM β-mercaptoethanol) on an ÅKTA system (GE Healthcare). Pfkinesin-8X-MD containing fractions were pooled and loaded onto a Superdex 200 Increase 10/300 GL gel filtration column (GE Healthcare) equilibrated with gel filtration buffer (20 mM PIPES pH 6.8, 80 mM KCl, 2 mM MgCl_2_, 1 mM ATP, 2 mM β-mercaptoethanol). Fractions containing monomeric protein were pooled and concentrated to around 50 μM using Amicon Ultra-0.5 ml Centrifugal Filters (Millipore), and then aliquoted and snap-frozen until further use.

### ATPase activity

#### MT preparation

Unlabeled porcine brain tubulin was purchased as a lyophilised powder (Cytoskeleton, Inc.) The protein was solubilized in BRB80 buffer (80 mM PIPES-KOH pH 6.8, 1 mM EGTA, 1 mM MgCl_2_) to approximately 10 mg/ml (tubulin dimer concentration). Reconstituted tubulin was polymerised at 5 mg/ml final concentration in the presence of 5 mM GTP at 37°C for 1 h. After this a final concentration of 1 mM paclitaxel (Calbiochem) dissolved in DMSO was added and the microtubules incubated at 37°C for another 1 h.

Free tubulin remaining after polymerization was removed by pelleting the MTs by centrifugation at 392,000 g, removing the supernatant and resuspending the MT pellet in BRB80 buffer. Protein concentration was determined post-centrifugation by a Bradford assay.

#### Experimental set-up

MT-stimulated kinesin ATPase activity was measured using a standard enzyme-coupled assay [69]. The assay was performed using 250 nM kinesin motor domain titrated with paclitaxel-stabilised MTs (0–6 μM) in 100 μl ATPase reaction buffer containing an ATP regeneration system (80 mM PIPES pH 6.8, 50 mM NaCl, 5 mM MgCl_2_, 1 mM EGTA, 5 mM phosphoenolpyruvate (PEP), 280 μM NADH, 12 U pyruvate kinase and 16.8 U lactate dehydrogenase). The ATP regeneration by pyruvate kinase is coupled to NADH depletion by lactate dehydrogenase in the conversion of PEP to lactate. NADH depletion was monitored by the decrease in absorbance at 340 nm in a SpectraMax Plus-384 plate reader every 30 s over 10 min at 37°C (Pbkinesin-8X-MD) and every 10 s for 1 h at 26°C (Pfkinesin-8X-MD) operated by SoftMax Pro 5 software. An adapted Michaelis-Menten equation was used for curve fitting of the ATPase data, which included an additional correction for V_0_, the ATPase rate when no MTs were present (measured basal rates: Pfkinesin-8X-MD = 0.93 ATP/s; Pbkinesin-8X-MD = 0.71 ATP/s). For Pfkinesin-8X-MD, fit R-squared = 0.95, for Pbkinesin-8X-MD, fit R-squared = 0.88.

### MT depolymerization assay

#### MT preparation

MTs containing 10% X-rhodamine-labelled and 10% biotin-labelled tubulin (Cytoskeleton) were polymerized with GTP, paclitaxel-stabilised as above and left at room temperature for 48 h before use in a TIRF assay.

#### Experimental set-up

Flow chambers for Total Internal Reflection Fluorescence (TIRF) microscopy were made between glass slides, biotin-PEG coverslips (MicroSurfaces Inc.), and double-sided tape. Chambers were sequentially incubated with: 1) blocking solution (0.75% Pluronic F-127, 5 mg/ml casein) for 5 min, followed by two washes with assay buffer (80 mM PIPES, 5 mM MgCl_2_, 1 mM EGTA, 1 mM DTT and 20 μM paclitaxel); 2) 0.5 mg/ml neutravidin for 2 min, followed by two washes with assay buffer (80 mM PIPES, 5 mM MgCl_2_, 1 mM EGTA, 1 mM DTT and 20 μM paclitaxel); 3) 1:100 dilution of X-rhodamine-labelled MT solution for 2 min, followed by two washes with assay buffer supplemented with 1 mg/ml casein; 4) 2.5 μM unlabelled kinesin-8X-MD proteins in assay buffer supplemented with 5 mM nucleotide (as indicated) and an oxygen scavenging system (20 mM glucose, 300 μg/ml glucose oxidase, 60 μg/ml catalase).

An Eclipse Ti-E inverted microscope was used with a CFI Apo TIRF 1.49 N.A. oil objective, Perfect Focus System, H-TIRF module, LU-N4 laser unit (Nikon) and a quad band filter set (Chroma). Movies were collected at room temperature under illumination at 561 nm for 30 min with a frame taken every 10 s with 100 ms exposure on a iXon DU888 Ultra EMCCD camera (Andor), using the NIS-Elements AR Software (Nikon). Where necessary, image drift was corrected using StackReg rigid body transformation. Depolymerisation rates were determined from kymographs using Fiji software. The assay was run in the presence of ATP, AMPPNP or apyrase as a no-nucleotide control. For each condition, data from two or more movies were analysed.

### MT gliding assay

#### MT preparation

MTs containing 10% X-rhodamine-labelled tubulin were polymerized with GTP, paclitaxel-stabilised as above and left for 48 h at room temperature before use in a TIRF assay. To prepare polar MTs to detect gliding directionality, long “dim” MTs were first polymerised by mixing X-rhodamine-labelled tubulin and unlabelled tubulin at a 1:9 ratio to a final concentration of 2 mg/ml. This mix was incubated at 37°C for 2 h in the presence of 0.5 mM GMPCPP. MTs were then pelleted by centrifugation at full-speed in a bench-top centrifuge for 15 min. To add bright plus end caps to the MTs X-rhodamine-labelled tubulin and unlabelled tubulin were mixed in a 1:1 ratio. The unlabelled tubulin in this reaction had been previously incubated with 1 mM N-ethyl maleimide (NEM) on ice for 10 min, followed by incubation with 100 mM beta-mercaptoethanol on ice for 10 min to block growth from the MT minus-end. This bright mix was pre-warmed then added to the polymerised long, dim MTs and incubated at 37°C for 15 min. MTs were pelleted by centrifugation again and resuspended in BRB80 with 40 μM Taxol.

#### SNAPf–tagged kinesin-8X-MD protein preparation

SNAPf–tagged kinesin-8X-MD proteins (20 μM) were biotinylated in 50 μl reaction volumes by incubating with 40 μM SNAP-biotin (NEB) at 4°C for 1.5 h. Proteins were purified from excess SNAP-biotin by size-exclusion chromatography on a Superdex 75 Increase 3.2/300 column using an ÅKTAmicro system (GE Healthcare) in gel filtration buffer (20 mM Tris-HCl pH 7.5, 250 mM NaCl, 5 mM MgCl_2_, 1 mM DTT). Peak fractions were pooled, snap frozen in liquid nitrogen, and stored at -80°C until use.

#### Experimental set-up

For MT gliding assays, flow chambers were treated and imaged as above except that in step 3) biotinylated kinesin8X-MD was added instead of MTs and in step 4) the reaction mixture contained 5mM ATP together with 10% X-rhodamine-MTs (or 10% polarity marked GMPCPP MTs to determine directionality) instead of kinesin-8X-MD proteins. Gliding assay movies were collected at room temperature for 10 min with 2 s interval. The gliding rates of single MTs were measured from kymographs using Fiji software.

### Generation of transgenic parasites

The C-terminus of kinesin-8X was tagged with GFP by single crossover homologous recombination in the parasite. To generate the kinesin-8X-GFP line, a region of the *kinesin-8* gene downstream of the ATG start codon was amplified using primers T1931 and T1932, ligated to p277 vector, and transfected as described previously [70]. The p277 vector contains the human *dhfr* cassette, conveying resistance to pyrimethamine. A schematic representation of the endogenous *kinesin-8X* locus (PBANKA_080590), the constructs and the recombined *kinesin-8X* locus can be found in S2 Fig.

The gene-deletion targeting vector for *kinesin-8X was* constructed using the pBS-DHFR plasmid, which contains polylinker sites flanking a *T*. *gondii dhfr/ts* expression cassette conferring resistance to pyrimethamine, as described previously [61]. PCR primers N1051 and N1052 were used to generate an 830 bp fragment of *kinesin-8X* 5′ upstream sequence from genomic DNA, which was inserted into *Apa*I and *Hin*dIII restriction sites upstream of the *dhfr/ts* cassette of pBS-DHFR. A 933 bp fragment generated with primers N1053 and N1054 from the 3′ flanking region of *kinesin-8X* was then inserted downstream of the *dhfr/ts* cassette using *Eco*RI and *Xba*I restriction sites. The linear targeting sequence was released using *Apa*I/*Xba*I. A schematic representation of the endogenous *kinesin-8X* locus (PBANKA_080590), the constructs and the recombined *kinesin-8X* locus can be found in S2F Fig. The oligonucleotides used to generate the mutant parasite lines can be found in S2B Table. *P*. *berghei* ANKA line 2.34 (for GFP-tagging) or ANKA line 507cl1 expressing GFP (for gene deletion) were transfected by electroporation described previously [71]. Briefly, electroporation was done using Amaxa^TM^ Human T cell Nucleofactor Kit (Lonza) and U033 programme on Amaxa biosystems. The electroporated parasites were mixed immediately with 100 μl of reticulocyte-rich blood from a phenylhydrazine (6mg/ml, Sigma) treated, naïve mouse, incubated at 37°C for 20 min and then injected intraperitoneally. From day 1 post-infection pyrimethamine (70 μg/ml, Sigma) was supplied in the drinking water for four days. Mice were monitored for 15 days and drug selection was repeated after passage to a second mouse. Resistant parasites were then used for cloning by limiting dilution and subsequent genotyping.

### Parasite genotype analyses

For the parasites expressing a C-terminal GFP-tagged kinesin-8X protein, diagnostic PCR was used with primer 1 (IntT193) and primer 2 (ol492) to confirm integration of the GFP targeting construct. For the gene knockout parasites, diagnostic PCR was used with primer 1 (IntN105) and primer 2 (ol248) to confirm integration of the targeting construct, and primer 3 (N105 KO1) and primer 4 (N105 KO2) were used to confirm deletion of the *kinesin-8X* gene.

### Parasite phenotype analyses

Blood containing approximately 50,000 parasites of the kinesin-8X-KO line was injected intraperitoneally (i.p.) into mice to initiate infections. Asexual stages and gametocyte production were monitored by microscopy on Giemsa stained thin smears. Four to five days post-infection, exflagellation and ookinete conversion were examined as described previously [70] with a Zeiss AxioImager M2 microscope (Carl Zeiss, Inc) fitted with an AxioCam ICc1 digital camera. To analyse mosquito transmission, 30–50 *Anopheles stephensi* SD 500 mosquitoes were allowed to feed for 20 min on anaesthetized, infected mice whose asexual parasitaemia had reached 15% and were carrying comparable numbers of gametocytes as determined on Giemsa stained blood films. To assess mid-gut infection, approximately 15 guts were dissected from mosquitoes on day 14 post-feeding and oocysts were counted on an AxioCam ICc1 digital camera fitted to a Zeiss AxioImager M2 microscope using a 63x oil immersion objective. On day 21 post-feeding, another 20 mosquitoes were dissected, and their guts and salivary glands crushed separately in a loosely fitting homogenizer to release sporozoites, which were then quantified using a haemocytometer or used for imaging. Mosquito bite back experiments were performed 21 days post-feeding using naive mice and blood smears were examined after 3–4 days.

### Electron microscopy

Mosquito midguts at 14-day post-infection were fixed in 4% glutaraldehyde in 0.1 M phosphate buffer and processed for electron microscopy as previously described (56). Briefly, samples were post-fixed in osmium tetroxide, treated en bloc with uranyl acetate, dehydrated and embedded in Spurr's epoxy resin. Thin sections were stained with uranyl acetate and lead citrate prior to examination in a JEOL1200EX electron microscope (Jeol UK Ltd).

### Culture and gradient purification of schizonts and gametocytes

Blood cells obtained from infected mice (days 4 to 5 post-infection) were placed in culture for 24 h at 37°C (with rotation at 100 rpm) and schizonts were purified the following day on a 60% v/v NycoDenz (in PBS) gradient, harvested from the interface and washed (NycoDenz stock solution: 27.6% w/v NycoDenz in 5 mM Tris-HCl, pH 7.20, 3 mM KCl, 0.3 mM EDTA). Purification of gametocytes was achieved using a protocol described as previously [72] with some modifications [73].

### Liver stage parasite imaging

For *P*. *berghei* liver stage parasites, 100,000 HeLa cells were seeded in glass-bottomed imaging dishes. Salivary glands of female *A*. *stephensi* mosquitoes infected with kinesin-8X-GFP parasites were isolated and disrupted using a pestle to release sporozoites, which were pipetted gently onto the seeded HeLa cells and incubated at 37°C in 5% CO_2_ in complete minimum Eagle's medium containing 2.5 μg/ml amphotericin B (PAA). Medium was changed 3 h after initial infection and once a day thereafter. For live cell imaging, Hoechst 33342 (Molecular Probes) was added to a final concentration of 1 μg/ml, and parasites were imaged at 24, 48, 55 h post-infection using a Leica TCS SP8 confocal microscope with the HC PL APO 63x/1.40 oil objective and the Leica Application Suite X software.

### Fixed Immunofluorescence Assay and DNA content analysis

The Pbkinesin-8X-GFP gametocytes were purified and activated in ookinete medium then fixed at 0 min, 1–2 min, 6–8 min and 15 min post-activation with 4% paraformaldehyde (PFA, Sigma) diluted in microtubule stabilising buffer (MTSB) for 10–15 min and added to poly-L-lysine coated slides. Immunocytochemistry was performed using primary GFP-specific rabbit monoclonal antibody (mAb) (Invitrogen-A1122; used at 1:250) and primary mouse anti-α tubulin mAb (Sigma-T9026; used at 1:1000). Secondary antibodies were Alexa 488 conjugated anti-mouse IgG (Invitrogen-A11004) and Alexa 568 conjugated anti-rabbit IgG (Invitrogen-A11034) (used at 1 in 1000). The slides were then mounted in Vectashield 19 with DAPI (Vector Labs) for fluorescence microscopy. Parasites were visualised on a Zeiss AxioImager M2 microscope fitted with an AxioCam ICc1 digital camera (Carl Zeiss, Inc).

### Deconvolution microscopy

High resolution imaging was performed using an AxioCam ICc1 digital camera fitted to a Zeiss AxioImager M2 microscope using a 63x oil immersion objective. Post-acquisition analysis was carried out using Icy software—version 1.9.10.0. Images presented are 2D projections of deconvoluted Z-stacks of 0.3 μm optical sections.

### Ookinete motility assay and DNA content analysis

The assay was performed using Matrigel (Corning) as described previously [74] with some modification. Ookinete cultures were added to an equal volume of Matrigel on ice, mixed thoroughly, dropped onto a slide, covered with a cover slip, and sealed with nail polish. The Matrigel was then allowed to set at 20°C for 30 min. After identifying a field containing ookinetes, time-lapse videos were taken at every 5 s for 150 cycles.

DNA content of ookinetes was analysed by fluorimetry after Hoechst nuclear staining as described previously using Image J [75].

### Quantitative Real Time PCR (qRT-PCR) analyses

RNA was isolated from different parasite stages including asexual, purified schizonts, gametocytes, ookinetes and sporozoites using an RNA purification kit (Stratagene). cDNA was synthesised using an RNA-to-cDNA kit (Applied Biosystems). Gene expression was quantified from 80 ng of total RNA using SYBR green fast master mix kit (Applied Biosystems). All the primers were designed using primer3 (Primer-blast, NCBI). Analysis was conducted using an Applied Biosystems 7500 fast machine with the following cycling conditions: 95°C for 20 s followed by 40 cycles of 95°C for 3 s; 60°C for 30 s. Three technical replicates and three biological replicates were performed for each assayed gene. The *hsp70* (PBANKA_081890) and *arginyl-t RNA synthetase* (PBANKA_143420) genes were used as endogenous control reference genes. The primers used for qPCR can be found in S2B Table.

### RNAseq analysis

Libraries were prepared from lyophilized total RNA using the KAPA Library Preparation Kit (KAPA Biosystems). Libraries were amplified for a total of 12 PCR cycles (12 cycles of [15 s at 98°C, 30 s at 55°C, 30 s at 62°C]) using the KAPA HiFi HotStart Ready Mix (KAPA Biosystems). Libraries were sequenced using a NextSeq500 DNA sequencer (Illumina), producing paired-end 75-bp reads.

FastQC (https://www.bioinformatics.babraham.ac.uk/projects/fastqc/), was used to analyze raw read quality, and based on this information, the first 11 bp of each read and any adapter sequences were removed using Trimmomatic (http://www.usadellab.org/cms/?page=trimmomatic). Bases with Phred quality scores below 25 were trimmed using Sickle (https://github.com/najoshi/sickle). The resulting reads were mapped against the *P*. *berghei* ANKA genome (v36) using HISAT2 (version 2–2.1.0), using default parameters. Uniquely mapped, properly paired reads were retained using SAMtools (http://samtools.sourceforge.net/), and PCR duplicates were removed by PicardTools MarkDuplicates (Broad Institute). Genome browser tracks were generated and viewed using the Integrative Genomic Viewer (IGV) (Broad Institute).

Raw read counts were determined for each gene in the *P*. *berghei* genome using BedTools (https://bedtools.readthedocs.io/en/latest/#) to intersect the aligned reads with the genome annotation. Differential expression analysis was done by use of R package DESeq2 to call up- and down-regulated genes. Gene ontology enrichment was done on PlasmoDB (https://plasmodb.org/plasmo/) with repetitive terms removed by REVIGO (http://revigo.irb.hr/).

### Statistical analysis

All statistical analyses were performed using GraphPad Prism 7 (GraphPad Software). For qRT-PCR, an unpaired t-test was used to examine significant differences between wild-type and mutant strains.

## Supporting information

S1 Fig Phylogenetic analysis of kinesins in apicomplexan. Phylogeny of detected kinesin protein sequences. Proteins with the suffix "-b[NNN]" were retrieved directly from Wickstead et al. [3], where NNN denote kinesin gene. Proteins with the suffix "-m[NNN]" were also detected by the reciprocal best BLAST approach (see Methods). Tree was produced using PhyML [76] with the LG+G+I+F model selected by SMS [77]. Branch support was evaluated with the Bayesian-like transformation of approximate likelihood ratio test (aBayes). Genetic distance shown below tree. Note that kinesin-15 is a paraphyletic group.
(TIF)

S2 Fig Generation and genotypic analysis of Pbkinesin-8X-GFP and *Δkinesin-8X* parasites. (A) Analysis of kinesin-8X transcript level by qRT-PCR during different stages of *P*. *berghei* life cycle. Mean ± SD. n = 3 independent experiments. (B) Schematic representation of the endogenous *pbkinesin-8X* locus, the GFP-tagging construct and the recombined *kinesin-8X* locus following single homologous recombination. Arrows 1 and 2 indicate the position of PCR primers used to confirm successful integration of the construct. (C) Diagnostic PCR of *kinesin-8X* and WT parasites using primers IntT193 (Arrow 1) and ol492 (Arrow 2). Integration of the kinesin-8X tagging construct gives a band of 2167 bp. Tag = kinesin-8X-GFP parasite line. (D) Western blot of kinesin-8X-GFP (~188 kDa) and WT-GFP (~27 kDa) protein to illustrate kinesin-8X-GFP in gametocyte stage. (E) Live cell imaging of kinesin-8X-GFP parasites during erythrocytic schizogony (F) Schematic representation of the endogenous kinesin-8x locus, the targeting knockout construct and the recombined kinesin-8X locus following double homologous cross-over recombination. Arrows 1 and 2 indicate PCR primers used to confirm successful integration in the kinesin-8X locus following recombination and arrows 3 and 4 indicate PCR primers used to show deletion of the kinesin-8X gene. (G) Integration PCR of the kinesin-8X locus in WT-GFP and *Δkinesin-8X* (Mut) parasites using primers INT N105 and ol248. Integration of the targeting construct gives a band of 1.5 kb. (H) qRT-PCR analysis of transcript in WT-GFP and *Δkinesin-8X* parasites. Mean ± SD. n = 3 independent experiments.
(TIF)

S3 Fig Localisation analysis of Pbkinesin-8X-GFP with kinetochore marker Ndc80-Cherry during sporogony. Live cell imaging showing that kinesin-8X-GFP (green arrow) is located next to Ndc80-Cherry (red arrow), a kinetochore marker, in oocysts stage (A), suggesting that it is not colocalizing with Ndc80 but is adjacent to it. It is clearer in sporozoites where kinesin-8X is enriched next to nucleus and Ndc80 (B).
(TIF)

S4 Fig Analysis of morphology, DNA content and motility of *Δkinesin-8X* ookinetes. (A) Morphology of ookinetes showing no difference in WT-GFP and *Δkinesin-8X* parasites. (B) Fluorometric DNA content (N) analysis of WT-GFP and *Δkinesin-8X* ookinetes, after Hoechst nuclear staining. Nuclear fluorescence intensity of WT-GFP or mutant parasites from 24 h cultures was measured using ImageJ software. Values are expressed relative to the average fluorescence intensity of haploid ring-stage parasites from the same slide and corrected for background fluorescence (Error bar ±SD; n = 3 independent experiments, >10 ookinetes were analysed for each experiment). (C) Representative frames from time-lapse videos of a WT-GFP and *Δkinesin-8X* ookinete in Matrigel. Red arrow indicates the apical end of the ookinetes. Bar  =  5 μm. Graph shows the quantitative data for motile ookinete for WT-GFP and *Δkinesin-8X*. (Error bar ±SD; n = 3 independent experiments, >20 ookinetes were analysed for each experiment).
(TIF)

S1 Table Phylogenetic analysis of kinesins in Apicomplexans. (XLSX)

S2 Table Kinesin-8X-GFP phenotype analysis (A) and oligonucleotides used in this study (B).
(XLSX)

S3 Table List of differentially expressed genes between Δ*kinesin-8X* and WT activated gametocytes. (XLS)

S1 Video Gliding motility of WT-GFP ookinetes. (AVI)

S2 Video Gliding motility of Δ*kinesin-8X* ookinetes. (AVI)
