# Supplementary figures and images for: Plasmodium kinesin-8X associates with mitotic spindles and is essential for oocyst development during parasite proliferation and transmission
Source: PLoS Pathog. 2019 Oct 10;15(10):e1008048. doi: 10.1371/journal.ppat.1008048 (PMC6786531; doi:10.1371/journal.ppat.1008048)

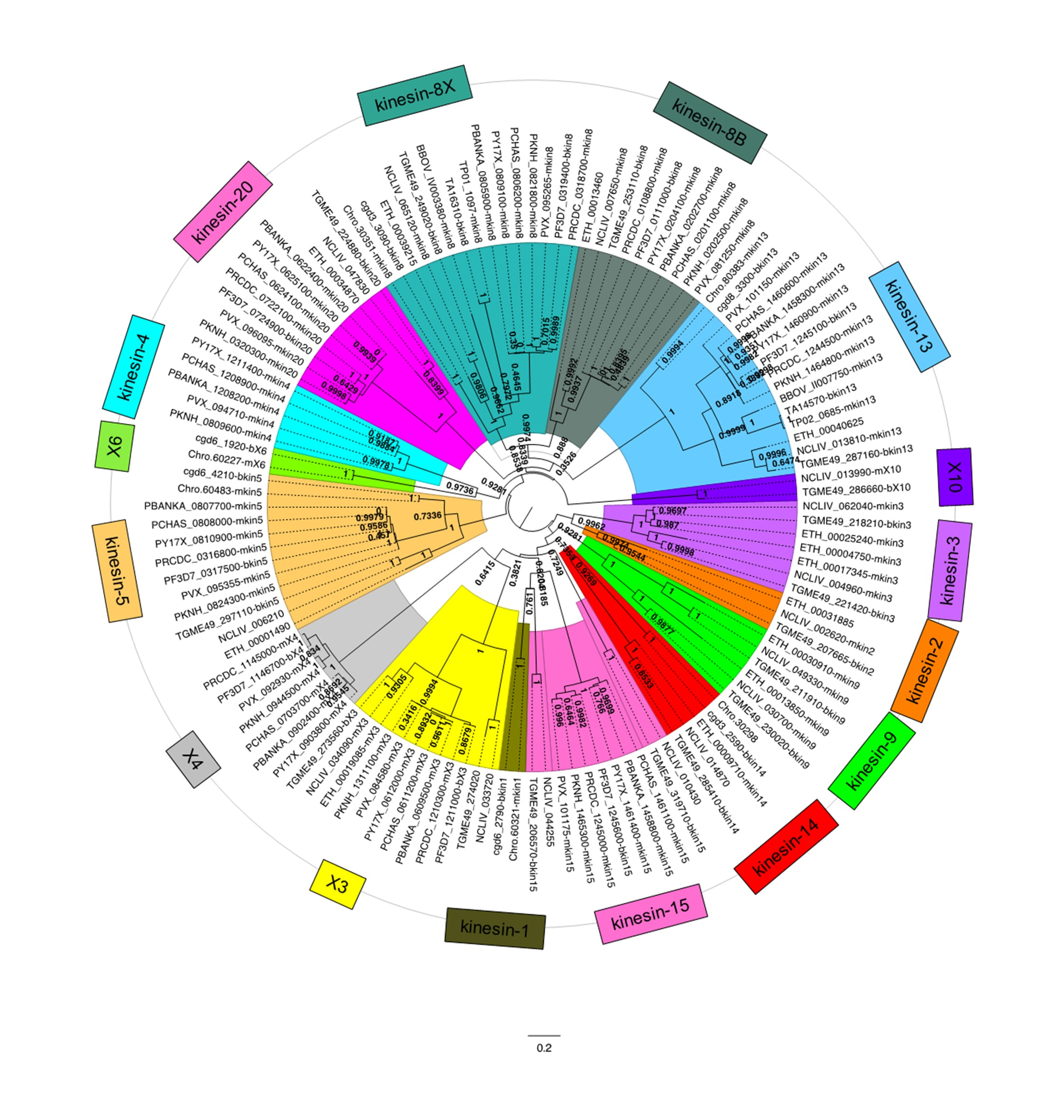

Supplement: S1 Fig — Phylogeny of detected kinesin protein sequences. Proteins with the suffix "-b[NNN]" were retrieved directly from Wickstead et al. [3], where NNN denote kinesin gene. Proteins with the suffix "-m[NNN]" were also detected by the reciprocal best BLAST approach (see Methods). Tree was produced using PhyML [76] with the LG+G+I+F model selected by SMS [77]. Branch support was evaluated with the Bayesian-like transformation of approximate likelihood ratio test (aBayes). Genetic distance shown below tree. Note that kinesin-15 is a paraphyletic group. (TIF) [file ppat.1008048.s001.tif]

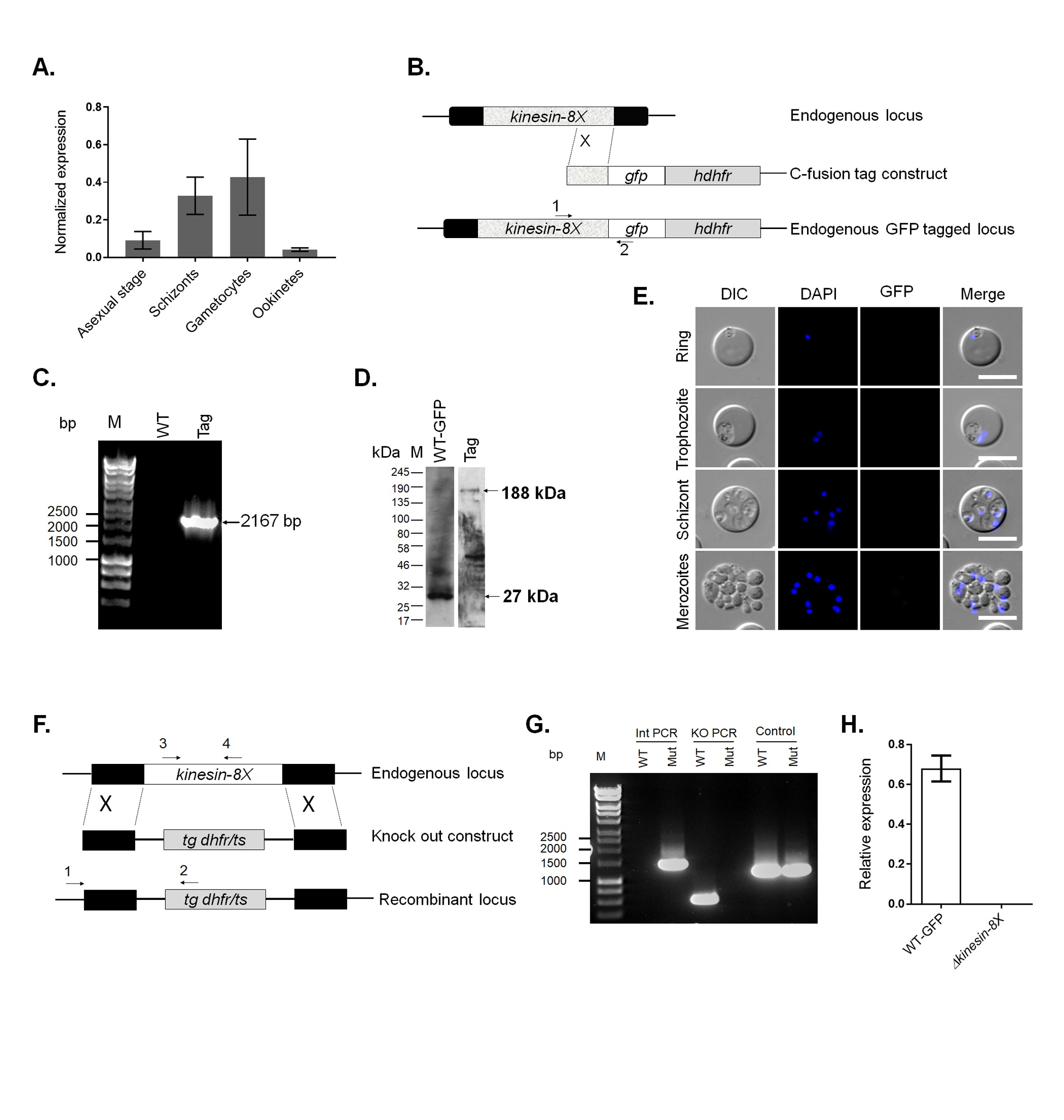

Supplement: S2 Fig — (A) Analysis of kinesin-8X transcript level by qRT-PCR during different stages of P. berghei life cycle. Mean ± SD. n = 3 independent experiments. (B) Schematic representation of the endogenous pbkinesin-8X locus, the GFP-tagging construct and the recombined kinesin-8X locus following single homologous recombination. Arrows 1 and 2 indicate the position of PCR primers used to confirm successful integration of the construct. (C) Diagnostic PCR of kinesin-8X and WT parasites using primers IntT193 (Arrow 1) and ol492 (Arrow 2). Integration of the kinesin-8X tagging construct gives a band of 2167 bp. Tag = kinesin-8X-GFP parasite line. (D) Western blot of kinesin-8X-GFP (~188 kDa) and WT-GFP (~27 kDa) protein to illustrate kinesin-8X-GFP in gametocyte stage. (E) Live cell imaging of kinesin-8X-GFP parasites during erythrocytic schizogony (F) Schematic representation of the endogenous kinesin-8x locus, the targeting knockout construct and the recombined kinesin-8X locus following double homologous cross-over recombination. Arrows 1 and 2 indicate PCR primers used to confirm successful integration in the kinesin-8X locus following recombination and arrows 3 and 4 indicate PCR primers used to show deletion of the kinesin-8X gene. (G) Integration PCR of the kinesin-8X locus in WT-GFP and Δkinesin-8X (Mut) parasites using primers INT N105 and ol248. Integration of the targeting construct gives a band of 1.5 kb. (H) qRT-PCR analysis of transcript in WT-GFP and Δkinesin-8X parasites. Mean ± SD. n = 3 independent experiments. (TIF) [file ppat.1008048.s002.tif]

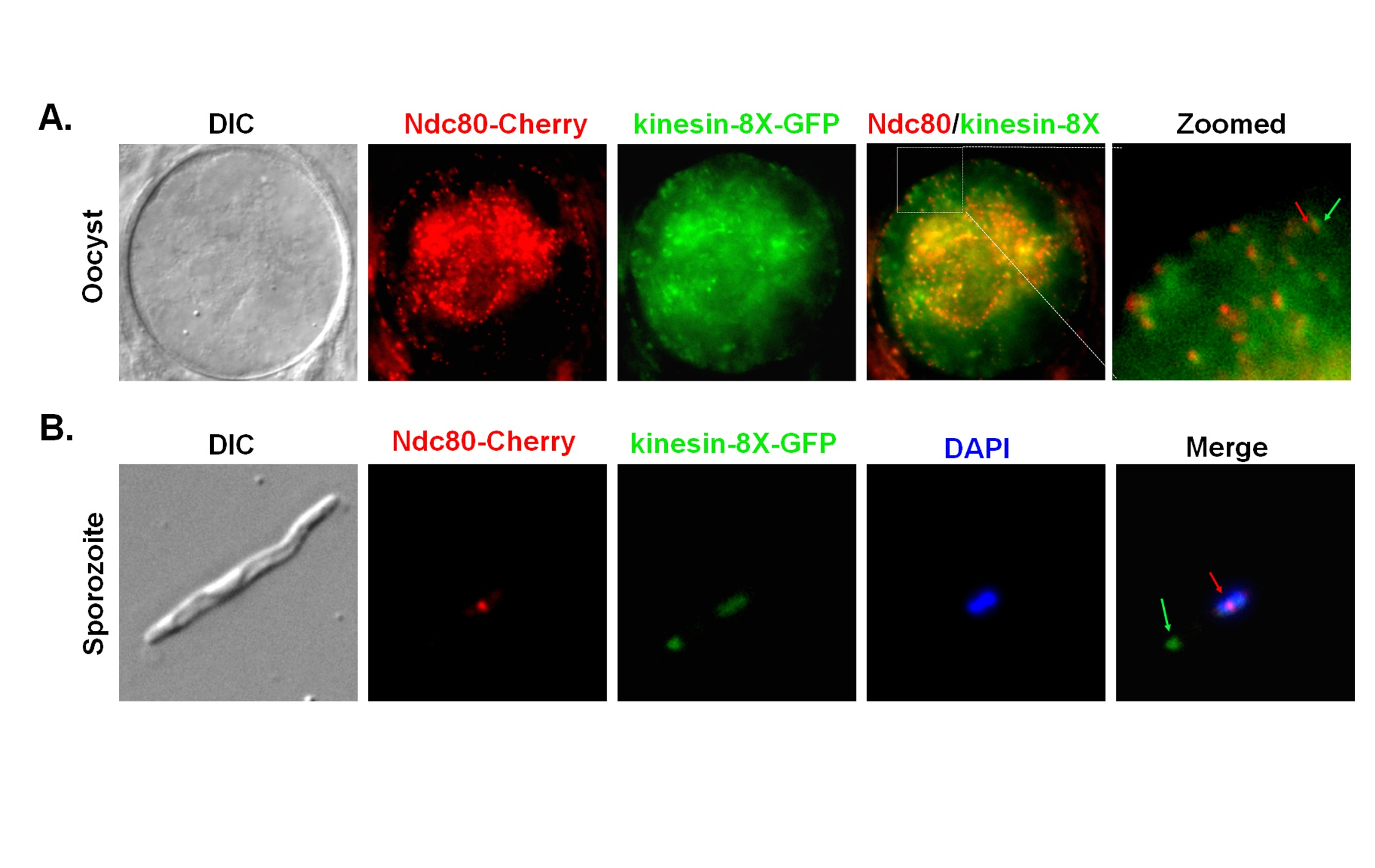

Supplement: S3 Fig — Live cell imaging showing that kinesin-8X-GFP (green arrow) is located next to Ndc80-Cherry (red arrow), a kinetochore marker, in oocysts stage (A), suggesting that it is not colocalizing with Ndc80 but is adjacent to it. It is clearer in sporozoites where kinesin-8X is enriched next to nucleus and Ndc80 (B). (TIF) [file ppat.1008048.s003.tif]

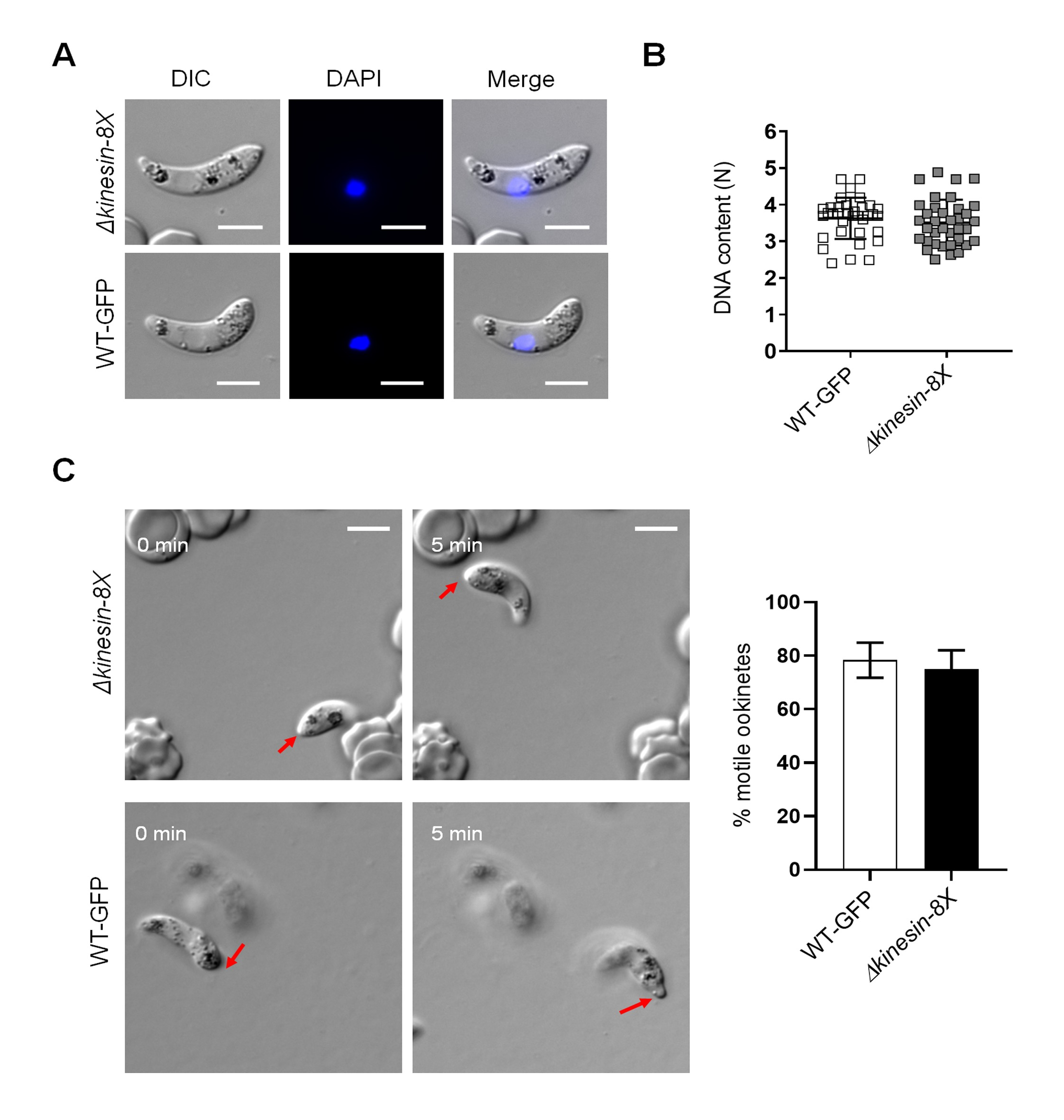

Supplement: S4 Fig — (A) Morphology of ookinetes showing no difference in WT-GFP and Δkinesin-8X parasites. (B) Fluorometric DNA content (N) analysis of WT-GFP and Δkinesin-8X ookinetes, after Hoechst nuclear staining. Nuclear fluorescence intensity of WT-GFP or mutant parasites from 24 h cultures was measured using ImageJ software. Values are expressed relative to the average fluorescence intensity of haploid ring-stage parasites from the same slide and corrected for background fluorescence (Error bar ±SD; n = 3 independent experiments, >10 ookinetes were analysed for each experiment). (C) Representative frames from time-lapse videos of a WT-GFP and Δkinesin-8X ookinete in Matrigel. Red arrow indicates the apical end of the ookinetes. Bar = 5 μm. Graph shows the quantitative data for motile ookinete for WT-GFP and Δkinesin-8X. (Error bar ±SD; n = 3 independent experiments, >20 ookinetes were analysed for each experiment). (TIF) [file ppat.1008048.s004.tif]
